# Dissecting Multivalent Carbohydrate Binding through Controlled Ligand Patterns on Cyclic Nanoscaffolds

**DOI:** 10.1021/acs.biomac.5c02630

**Published:** 2026-04-24

**Authors:** Xin-Yu Wang, Zong-You Lee, Meng-Che Li, Masayuki Hashimoto, Kwun-Yung Cheung, Yi-Tsu Chan, Wei-Chieh Cheng, Sheng-Kai Wang

**Affiliations:** † Department of Chemistry, 34881 National Tsing Hua University, Hsinchu 300044, Taiwan; ‡ Institute of Molecular Medicine, College of Medicine, 34912 National Cheng Kung University, Tainan 701401, Taiwan; § Department of Chemistry and Center for Condensed Matter Sciences, 33561 National Taiwan University, Taipei 106319, Taiwan; ∥ Genomics Research Center, 38017 Academia Sinica, Taipei 115024, Taiwan

## Abstract

Carbohydrate–protein interactions are essential for biological recognition but often suffer from cross-reactivity. Multivalency can enhance the binding strength; however, it requires a precise spatial arrangement of carbohydrate ligands to match the protein binding sites. Controlling glycan presentation improves both avidity and selectivity, helping to reduce cross-reactivity. However, complex proteins, such as AB5-type Shiga toxin (Stx), present additional challenges, as the B subunits form pentamers, and each subunit contains three nonequivalent glycan-binding sites. To address this, we developed oligoproline-based cyclic nanoscaffolds and characterized them by using circular dichroism and ion-mobility spectrometry. Surface plasmon resonance analysis showed that different glycan patterns on these scaffolds produced distinct binding modes with the StxB pentamer. By coupling a fully tunable synthetic nanoscaffold platform with analytical methods capable of resolving complex binding behaviors, this work enables a deeper investigation of protein receptors and supports the design of more selective multivalent biomolecules.

## Graphical abstract

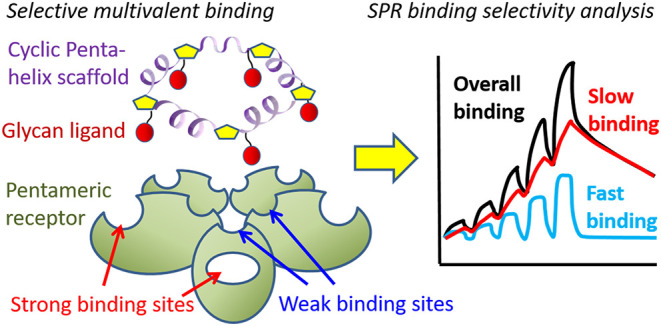

## Introduction

Carbohydrate–protein interactions play essential roles in biological recognition, including cell–cell interactions and pathogen infection, and they are consequently considered a promising approach to manipulate the related biological processes.^1^ Despite the generally weaker monomeric carbohydrate–protein binding strength compared to protein–protein interactions, the simultaneous binding of multiple carbohydrate ligands to an oligomeric protein significantly enhances the overall binding strength.^2^ In addition, the typical problem of carbohydrate-binding cross-reactive recognition is reduced by multivalent interactions, as it requires spatial matching of the ligand presentation to the arrangement of binding sites on the protein oligomer to achieve a strong interaction, which adds extra selectivity.^3–5^ Therefore, governing multivalent carbohydrate interactions can be crucial for intervening in related pathogen infections and other biological events.

One challenge in designing multivalent carbohydrate ligands is how to properly present the ligands when multiple binding sites on the target protein have a specific arrangement, wherein the sites are typically a few nanometers apart. The traditional use of branched long flexible linkers to connect ligands may cause an entropy penalty^2^ and cross-reactivity problem, as the ligands can adapt their pattern to match various nontarget protein oligomer arrangements.^6^ In some cases, multivalent binding is even more complicated when the target proteins have heterobinding sites with different binding activities within a single subunit. Designing appropriate multivalent ligand presentations for such complex proteins or evaluating the different binding modes between ligands and these proteins poses significant challenges for glycoscientists.

Bacterial Shiga toxins (Stx) are found in *Shigella dysenteriae* and Shiga toxin-producing *E. coli* (STEC), and they can cause diarrhea and may lead to life-threatening hemolytic-uremic syndrome (HUS).^7^ These toxins belong to the AB5 toxin family, which has five pentagonally arranged B subunits that bind to cell surface ligands to deliver the toxic A subunit (Figure 1a) into the host cell and traffick to the endoplasmic reticulum through retrograde transport.^8^ The A subunit for Shiga toxin is an RNA N-glycosidase that depurinates a specific rRNA nucleotide to terminate ribosome translation^9^ and lead to cell death.^10^ However, without the help of B subunits, the A subunit cannot enter the host cell, so inhibition of the B subunits can reduce the cytotoxic effect. In addition, the delicate and efficient delivery system of B pentamers has been exploited for many promising biomedical applications, such as tumor imaging, cancer drugs, drug delivery, and microarrays.^11^ The Stx B pentamer functions by binding to the trisaccharide of globotriaosylceramide (Gb3 ceramide) (Figure 1b), a glycolipid on the cell surface.^12^ Further binding analysis and structural studies have shown that there are three globotriose (Gb3) binding sites per Stx B subunit, giving a total of 15 sites in each Stx complex.^13^ Because of the key role that the Stx B pentamer plays in toxin function and biomedical applications, it is essential to investigate the multivalent binding of the Gb3 trisaccharide to this protein oligomer for more precise targeting. With this aim in mind, two of the core challenges are as follows: (1) how to precisely present multiple ligands toward the desired binding site on each Stx B subunit and (2) how to evaluate multivalent binding among Stx B major and minor binding sites.

**Figure 1:**
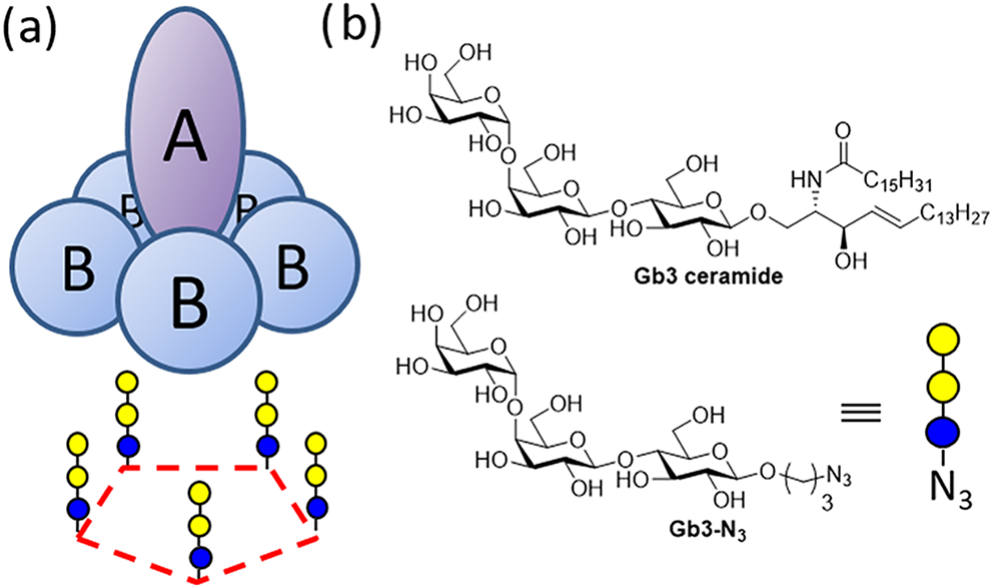
Arrangement of Shiga toxin subunits and matching multivalent ligand presentation (a). Glycan ligand structures for Shiga toxin (b).

## Experimental Section

### Materials

The reagents for peptide synthesis were purchased from Sigma-Aldrich, Acros, Alfa Aesar, and Thermo Fisher in ACS grade. The 2-chlorotrityl chloride resins for SPPS were purchased from Merck (Product No. 855017). The reagents for organic synthesis and bioconjugation were purchased from Sigma, Merck, and TCI in ACS grade. An SPR sensor chip CM5 was purchased from GE Healthcare.

### General Solid-Phase Peptide Synthesis

The peptides were prepared by manual solid-phase peptide synthesis on 2-chlorotrityl chloride resin. A solution of Fmoc-Pro-OH (4.0 equiv) and DIEA (6.0 equiv) in 1/1 (v/v) DMF/DCM (final concentration, 0.1 M) was added to the resins. The mixture was gently shaken overnight and washed with DMF × 3, DCM × 3, and DMF × 3. A solution of DCM/MeOH/DIPEA (v/v/v = 17:2:1) was added, and the mixture was shaken for 1 h to block unreacted sites on the resins. After washing with DMF × 3, DCM × 3, and DMF × 3, the Fmoc-Pro-OH loading was determined with a quantitative Fmoc test with a UV spectrometer.

The resins were further used for iterative peptide synthesis. 10% piperidine in DMF (enough to cover the resins) was added to the resins twice to deprotect the Fmoc group. Each time, the reaction vessel was shaken for 10 min and washed with DMF × 3, DCM × 3, and DMF × 3. Then, a mixture of amino acids (4.0 equiv, Fmoc-Pro-OH or *N*-Fmoc-*trans*-4-(N-benzyloxycarbonyl)­amino-l-proline based on the designed sequence) and HATU (4.0 equiv) was dissolved in DMF, NMM was added (4.0 equiv), and the mixture was reacted with deprotected resins (final concentration, 0.05 M). The mixture was gently shaken for 1 h and then washed with DMF × 3, DCM × 3, and DMF × 3.

After each coupling step, the resins were treated with Ac_2_O/pyridine (1:9, enough to cover the resins) and shaken for 10 min to cap the unreacted amino groups. The resins were washed with DMF × 3, DCM × 3, and DMF x 3 and then used for the next round of synthesis. To cleave the peptide, the resins were washed with DMF × 3 and DCM × 3, treated with DCM/TFA/TIS (90:5:5, enough to cover the resins), and shaken for 1 h. This process was repeated for a second time.

The filtrate was collected, and all the volatiles were removed by rotary evaporation under reduced pressure. Water was added to the resultant residue and centrifuged before the supernatant was purified by HPLC (Agilent Technology, 1200 Infinity and 1260 Infinity) with a Vydac C18 column (218TP54 250 mm × 4.6 mm) and Vydac C18 column (218TP10510 250 mm × 10 mm) operating at a flow rate of 0.5 or 3 mL/min using a mobile phase of solvent A (0.1% TFA in H_2_O) and solvent B (acetonitrile). The mass of the peptides was confirmed by matrix-assisted laser desorption ionization-time-of-flight (MALDI-TOF) mass spectrometry (Bruker Daltonics, Autoflex III smartbeam LRF200-CID).

### Peptide C-Terminus Alkyne Modification

To a solution of peptide acid (1.0 equiv) obtained from SPPS and HATU (4.0 equiv) dissolved in DMF/CH_2_Cl_2_ (1:1, peptide concentration 0.02 M), propargylamine (3.0 equiv) and Et_3_N (5.0 equiv) were added. After the mixture was stirred for 1 h, CH_2_Cl_2_ was removed from the reaction mixture under reduced pressure. The remaining mixture was purified by HPLC with a Vydac C18 column to obtain peptide **1**.

### Peptide N-Terminus Azido Modification

10% piperidine in DMF (enough to cover the resins) was added to the resins 2 times to deprotect the Fmoc group. Each time, the vessel was shaken for 10 min and washed with DMF × 3, DCM × 3, and DMF × 3.

To a solution of azido linker 4-azidobutanoic acid succinimidyl ester (8 equiv) in DMF, DIPEA was added (16 equiv) and reacted with the resins carrying N-terminus deprotected peptide in DMF (final concentration of azido linker at 0.05 M). The reaction vessel was gently shaken for 1 h, and then the resins were washed with DMF × 3, DCM × 3, and DMF × 3.

### Solid-Phase Peptide Ligation

The azido-functionalized peptide on resins was treated with a mixture of alkynyl peptide **1** (1.5 equiv), CuSO_4_ (aq) (0.13 equiv, 40 mM), tris­(triazoly)­amine ligand **2** (0.13 equiv, 40 mM in DMSO), sodium ascorbate (aq) (2.6 equiv, 800 mM), and DIPEA (4.0 equiv) in THF (final copper concentration 2 mM) for 16 h. The resultant resins were washed with sodium (diethylcarbamothioyl)­sulfanide solution (5 mg in 1 mL of DMF with 5 μL of DIPEA) × 5, DMF × 3, DCM × 3, and DMF × 3.

### Peptide Cyclization

Linear peptide tetramer **3** and pentamer **4** were dissolved in water at 2 mM, and CuSO_4_ (aq) (0.13 equiv, 40 mM), tris­(triazoly)­amine ligand (**2**) (0.13 equiv, 40 mM in DMSO), sodium ascorbate (aq) (2.6 equiv, 800 mM), and DIPEA (4.0 equiv) were added and reacted at room temperature for 1 h. The product was purified by HPLC with a Vydac C18 column to obtain cyclized peptides **5** (34%) and **6** (37%).

### *N*-Cbz Deprotection of Cyclic Peptides

To the solution of Cbz-protected cyclic peptides **5** and **6** dissolved in MeOH (peptide concentration of at least 0.4 mM) in a round-bottom flask, formic acid (1:10 (v/v) of total volume) and Pd­(OH)_2_ on carbon (four times the weight of the peptide) were added. The solution was stirred under a hydrogen atmosphere for 16 h. The resultant reaction mixture was centrifuged to remove palladium hydroxide on carbon and washed with MeOH several times. The combined solution was concentrated under reduced pressure. The crude product was dissolved in H_2_O and purified by HPLC with a Vydac C18 column.

### Alkyne Linker Installation on Cyclic Peptide Scaffolds

To the solution of Cbz-deprotected amino peptide in DMF/DCM (peptide concentration, 2 mM) in a microcentrifuge tube, HATU (5 equiv for each reacting site) was added, and the mixture was shaken for 10 min. To the reaction mixture, then 4-pentynoic acid or 2-(2-(prop-2-yn-1-yloxy)­ethoxy)­acetic acid **15** (20 equiv) and DIPEA (40 equiv) were added, and the mixture was shaken at room temperature for 3 h. The resultant solution was concentrated under reduced pressure to remove DCM and purified by HPLC with a Vydac C18 column to obtain cyclic peptides **7**–**10**.

### Glycan Conjugation on Alkynyl Cyclic Peptide Scaffolds

To a solution of alkynyl cyclic peptide scaffolds **7**, **8**, **9**, or **10** in H_2_O (peptide concentration 1 mM) in a microcentrifuge tube, Gb3-N_3_ (50 equiv) was added, and the mixture was shaken for 10 min. To the reaction mixture, a solution of CuSO_4_ (aq) (50 equiv, final 10 mM), tris­(triazoly)­amine ligand **2** in DMSO (50 equiv, final 10 mM), and sodium ascorbate (aq) (750 equiv, final 150 mM) was added. The reaction mixture was shaken at 40 °C for 2 h. The resultant solution was concentrated under reduced pressure and purified by HPLC with a Vydac C18 column to obtain peptides **11**, **12**, **13**, and **14**.

### Traveling Wave Ion Mobility Spectrometry (TWIM)-Mass Spectrometry Analysis

Mass spectrometry and TWIM experiments were conducted on a Waters Synapt HDMS G2 instrument with a LockSpray ESI source,^14^ using the following parameters: ESI capillary voltage, 3.0 kV; sample cone voltage, 40 V; extraction cone voltage, 0.0 V; desolvation gas flow, 500 L h^–1^ (N_2_); source temperature, 100 °C; and desolvation temperature, 300 °C. For the TWIM experiments, the helium cell gas flow was maintained at 180.0 mL min^–1^, and the ion mobility cell gas flow was maintained at 90.0 mL min^–1^ (N_2_). The DC traveling wave velocity and height were set at 683 m s^–1^ and 26.3 V, respectively. All samples were dissolved in H_2_O to a concentration of 0.3 mM and then infused into the ESI source at a flow rate of 5 μL min^–1^ with a syringe pump (KDS-100, KD Scientific). Data were collected and analyzed by using MassLynx 4.1 and DriftScope 2.4 (Waters).

The experimental calibration curve of the collision cross section (CCS) was established according to a previously reported protocol,^15^ and the CCS values in the He drift gas were used for calibration. A plot of the corrected drift times versus the corrected cross sections of the calibrants fitted with power functions was used as a calibration curve for the CCS measurements.

### Preparation of Stx1B^16^

The gene-encoding stx1B from *E. coli* O157:H7 EDL933 was cloned into the pET28 vector to add a 6xHis tag at its C-terminus. A fragment encoding the gene was amplified by PCR with EDL-stx1B-1 (5′-GGGAGAGCCATGGCGACGCCTGATTGTGTA-3′) and EDL-stx1B-2 (5′-AGAGGGATCCGCACGAAAAATAACTTCGCTGA-3′) from the EDL933 genome and then cloned into the *Nco*I and *Bam*HI sites of pET28c. *E. coli* BL21­(DE3) harboring the plasmid was cultured in 200 mL of LB broth supplemented with kanamycin (50 μg/mL) and IPTG (1 mM) for 6 h. The cells expressing Stx1B were lysed by sonication, and the protein was purified with HisTrap FF (1 mL, Cytiva). The fractions containing purified Stx1B were pooled and applied to an Amicon Ultra Centrifugal filter (3 kDa MWCO, Millipore) to exchange the buffer with phosphate-buffered saline (PBS). The purified protein, which had a calculated molecular mass of 10 371 Da (monomer), was observed as a single band on SDS-PAGE and stored at −20 °C.

### SPR Analysis

Surface plasmon resonance (SPR) experiments were performed on a Biacore T200 at 25 °C using a functionalized CM5 sensor chip. Protein immobilization was performed according to the built-in wizard software template of the instrument. Stx1B immobilization assays were performed with immobilization buffer PBS (137 mM NaCl, 2.7 mM KCl, 10 mM Na_2_HPO_4_, 1.8 mM KH_2_PO_4_, pH 7.4).^17^ The CM5 sensor chip was activated using a solution containing *N*-ethyl-*N* ^*’*^-(3-diethyl-aminopropyl)-carbodiimide (EDC) (0.2 M) and *N*-hydroxysuccinimide (NHS) (0.05 M). Stx1B (20 μg/mL) in acetate buffer (pH 5.0, 10 mM) was injected over the activated surface at a flow rate of 10 μL/min for 420 s. Then, ethanolamine (pH 8.5 and 1 M) was injected to block the remaining activated groups.

Binding assays were performed with PBS buffer (300 mM NaCl, 2.7 mM KCl, 10 mM Na_2_HPO_4_, 1.8 mM KH_2_PO_4_, pH 7.4, 0.005% TWEEN 20) as the running buffer. Glycoconjugates **11–14** and Gb3-N_3_ were subjected to single-cycle kinetics analyses (analyte contact time of 120 s for each concentration, 60 s running buffer between two concentrations of analyte, dissociation time of 600 s, flow rate of 20 μL/min) on the surface, with concentrations of 125, 250, 500, 1000, and 2000 nM for **11** and **13**; 31.25, 62.5, 125, 250, 500 nM for **12** and **14**; and 62.5, 125, 250, 500, 1000 μM for Gb3-N_3_ diluted in the running buffer. The surface had at least 688 RU Stx1B immobilized, and the surface was regenerated by a 30 s injection of regeneration buffer (100 mM methyl-β-d-galactopyranoside, pH 7.8). The sensorgrams were reference subtracted, quality controlled, and analyzed by the Biacore T200 Evaluation Software. The kinetic parameters were obtained by fitting curves to a 1:1 Langmuir model and a heterogeneous ligand model.

## Results and Discussion

To effectively control multiple ligand positions at the nanoscale, it is essential to use a suitable nanoscaffold to support multiple ligands. A rigid scaffold can reduce conformational deformation to limit ligand displacement from the desired positions, thereby mitigating the cross-reactivity to nontarget proteins that comes with alternative binding site arrangements. We have previously developed a cyclic nanoscaffold system by connecting three oligoproline peptides into a macrocyclic nanoscaffold to control ligand presentation.^18^ In an aqueous environment, the oligo/polyproline peptides have a stable and unique helical conformation known as polyproline helix II (PPII), in which every proline residue rotates about 120° from the previous one to give a helical pitch of about 0.9 nm.^19,20^ Having such structural features and biocompatibility, the PPII peptides have been exploited for various applications in chemical biology.^21–24^ We have exploited the polyproline oligo-helix macrocyclic (PPnM) scaffolds to manipulate multivalent carbohydrate–lectin binding^18^ and also demonstrated the excellent lectin selectivity on DC-SIGN/langerin compared to a traditional glycodendron scaffold, which has inadequate spatial control of ligand positions due to the use of a flexible structure and long linkers.^6^

### Scaffold Design and Synthesis

To target pentameric Stx1B, we designed an unprecedented polyproline penta-helix macrocycle (PP5M) scaffold by connecting five Pro_6_ peptides and compared it to its tetra-helix analog (PP4M) for different Gb3 glycan presentations (Scheme 1). The syntheses of PP5M and PP4M scaffolds were conducted with the following strategy: Briefly, the Pro_6_ peptide was prepared through solid-phase peptide synthesis (SPPS) on 2-Cl-trityl chloride resins, and a Cbz-protected amine group was installed at C-4 of the N-terminal proline. The Fmoc-protected Pro_6_ peptide cleaved from a portion of the resin was modified with an alkyne group at the C-terminus (peptide **1**) and further ligated with the resin-bound azido-Pro_6_ via the CuAAC reaction.^25^ The repeated solid-phase ligation cycle, composed of Fmoc deprotection, azido linker installation, and CuAAC ligation, allowed us to efficiently synthesize oligo-helix peptides containing the desired number of helices in the desired order. In this manner, linear tetra- and penta-Pro_6_ peptides were synthesized, cleaved from resins, and alkyne groups were installed at the C-termini as peptides **3** and **4**, respectively. The cyclization of linear peptides **3** and **4** was achieved through another CuAAC reaction to yield PP4M (**5**) and PP5M (**6**) scaffolds with Cbz-protected amines at the first position of each Pro_6_ helix.

**Scheme 1:**
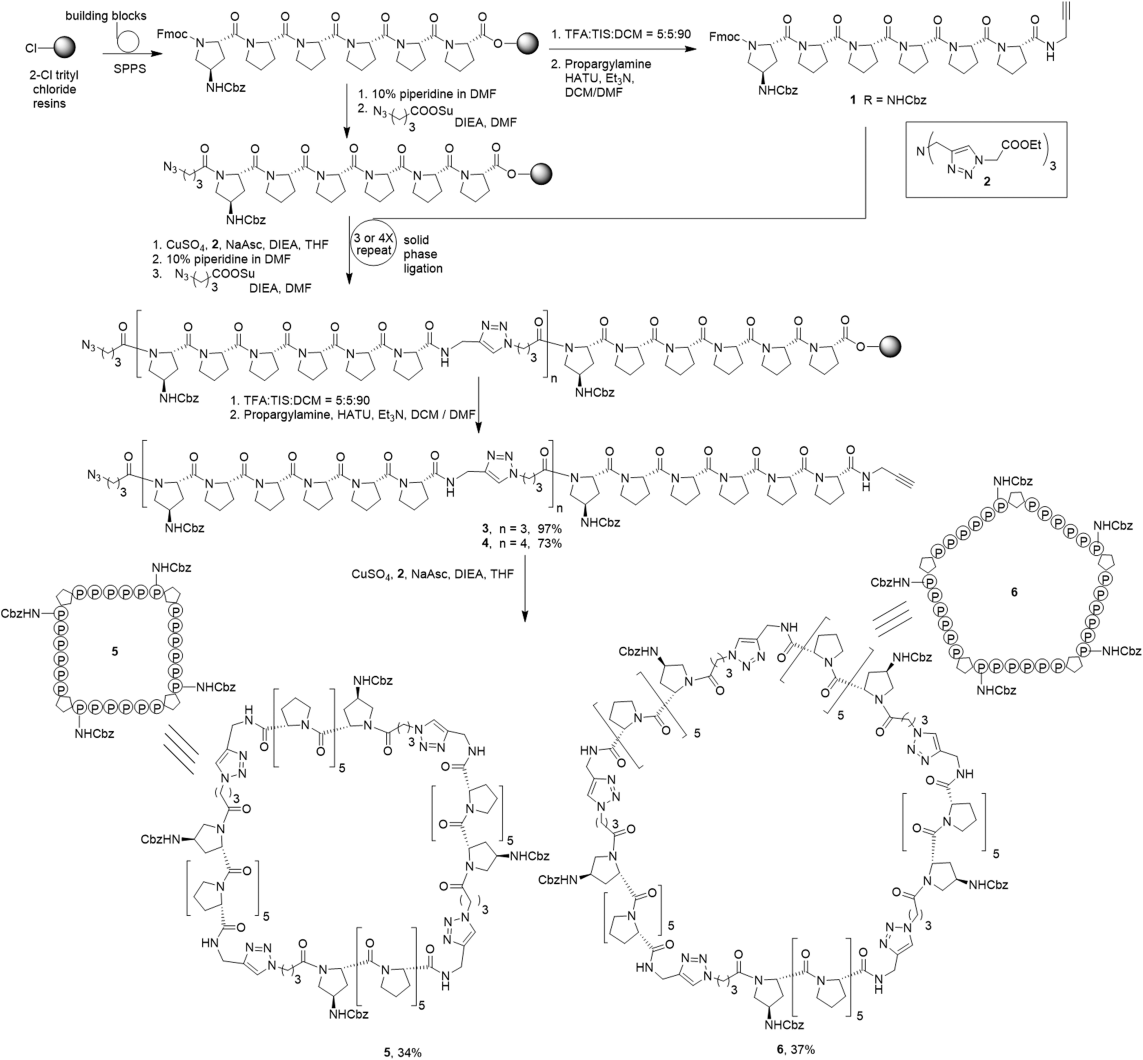
Synthesis of Cyclic Nanoscaffolds **5** and **6**

### Circular Dichroism Analysis

As this was the first time a PP5M-type scaffold had been synthesized, we characterized the peptide conformation with circular dichroism (CD) spectroscopy. The oligo/polyproline peptide typically transits between PPII and PPI conformations, which have opposite handedness in their helix structures.^26^ These conformations can be identified by the λ_max_ of the CD spectra, as well as the ratio of the maximal positive intensity to the maximal negative intensity (*R*_pn_).^27^ In addition, comparing the CD spectra between linear and cyclized scaffolds also provides information regarding the cyclic strain.^18,28^ We measured the CD spectra of linear scaffolds **3**–**4** and cyclized scaffolds **5**–**6** in both water and *n*-propanol, as shown in Figure 2, and the parameters are listed in Table 1. The CD measurements for these peptides in water showed all their λ_max_ values to be around 228 nm (Table 1 and Figure 2a,b), with the minimum at around 205 nm, clearly showing typical PPII characteristics.^29,30^ These findings essentially allowed us to estimate the dimensions of the cyclic scaffold on the basis of the well-defined PPII helical structure.

**Figure 2:**
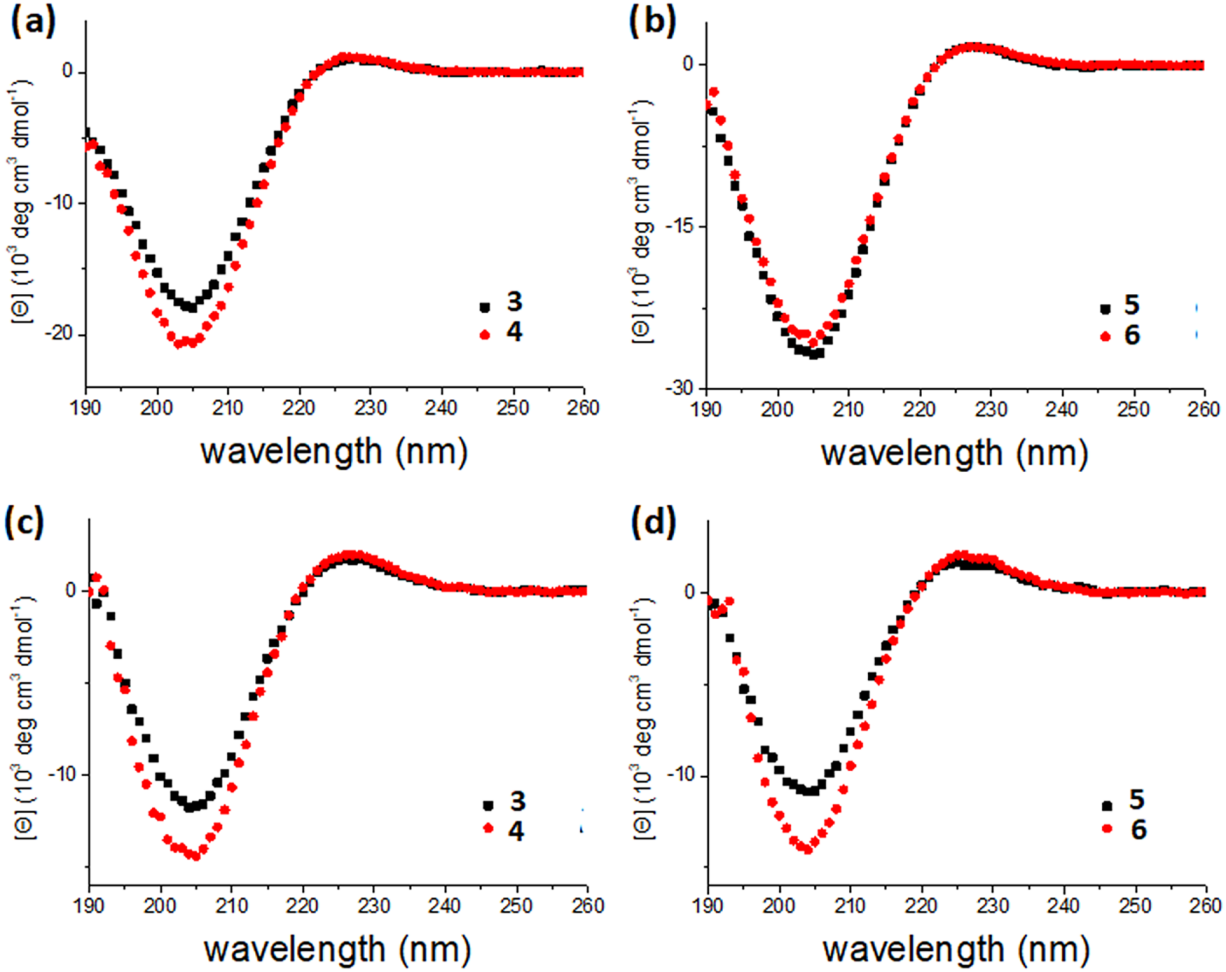
CD spectra of scaffolds **3**–**6**. Linear scaffolds **3**–**4** measured in water (a) and *n*-propanol (c); cyclized scaffolds **5**–**6** measured in water (b) and *n*-propanol (d). All samples were measured at 100 μM.

**Table 1:** CD Spectra Parameters of Scaffolds **3–6** Measured at 100 μM

	in H_2_O			in *n*-propanol		
scaffold type	λ_max_ (nm)	λ_min_ (nm)	*R*_pn_ ^a^	λ_max_ (nm)	λ_min_ (nm)	*R*_pn_ ^a^
linear (Pro_6_)_4_ **3**	228	205	0.062	228	204	0.152
linear (Pro_6_)_5_ **4**	226	203	0.058	228	205	0.140
cyclic (Pro_6_)_4_ **5**	227	205	0.064	225	204	0.151
cyclic (Pro_6_)_5_ **6**	227	205	0.066	226	204	0.149

a Represents the ratio of the positive peak intensity to the negative peak intensity.

Unlike in an aqueous environment, the CD spectra of polyproline peptides in *n*-propanol typically favor the PPI conformation,^31–33^ but they may also be affected by other factors, such as the substituents on proline residues,^34,35^ charges at the peptide termini,^36^ and the length of the peptide.^37^ The measured CD spectra of scaffolds **3**–**6** in *n*-propanol also showed the characteristics of the PPII conformation (Figure 2c,d), likely due to the shorter Pro_6_ peptide helices in combination with the stereoelectronic effect of the *trans* Cbz-protected amine substituent. Thus, although a higher PPI propensity is reflected by a higher *R*_pn_ in *n*-propanol (0.064–0.151 and 0.066–0.149, Table 1), this solvent effect is not enough, leading to a fully PPI conformation. In addition to determining the peptide conformation, we have previously observed significant changes in *R*_pn_ measured in *n*-propanol from the increased ring strain upon cyclization of certain trihelix scaffolds.^28^ However, in the present work, it is not surprising that the cyclization of **3** and **4** to **5** and **6** did not lead to meaningful changes in *R*_pn_ (0.152–0.151 and 0.140–0.149, Table 1), as Pro_6_ is too short to cause significant changes in CD spectra and the cyclization of tetra-helix and penta-helix scaffolds is expected to generate less ring strain than the previous trihelix counterparts. Combining these observations, cyclized scaffolds **5** and **6** remain in the PPII conformation in aqueous solution and have no significant ring strain.

### Traveling Wave Ion Mobility Mass Spectrometry Analysis

In addition to CD spectroscopy, we also analyzed linear and cyclized nanoscaffolds **3**–**6** with Traveling Wave Ion Mobility–Mass Spectrometry (TWIM-MS). TWIM-MS combines ion mobility analysis, which separates gas molecules by their shape and size, with mass spectrometry, which determines the identity of the received signal. This technology has become an excellent approach for macromolecule analysis, as it allows the ionized analyte under an electric field to fly through a drift chamber containing trace buffer gas molecules for collision, which delays the analyte ion detection using a mass spectrometer. The measured delay, also known as drift time, is responsive to the collision probability and charge carried by the analyte ion. Therefore, based on TWIM-MS measurements, the collision cross section (CCS) of an analyte can be estimated to provide structural information.

Representative TWIM-MS results for **6** are shown in Figure 3, and the rest of the data can be found in the Supporting Information. Three major groups of cyclized scaffold **6** ions were observed, carrying 3, 4, and 5 positive charges, and they exhibited distinct drift times (Figure 3a). For each group, the observed isotope pattern matched well with the calculated one to confirm the identity of the measured ion, and the drift time distribution for each ion is also presented (Figure 3b,c and Supporting Information). The parameters obtained from the TWIM-MS analysis of scaffolds **3**–**6** (Table 2) indicated that the pentameric scaffolds had longer drift times than the corresponding tetrameric scaffolds of the same charge and style (linear or cyclic) to reflect their larger size. It is also noteworthy that the reduction in drift times in general responded to the scaffold cyclization from the linear (**3** and **4**) to the cyclized (**5** and **6**) forms. These results indicate that the linear oligo-helix scaffolds have a high probability of colliding with buffer gas molecules, similar to other cyclic/linear peptides that have been reported,^38,39^ possibly due to the loss of degrees of freedom from macrocyclization.^40^ From another parameter, the full width at half-maximum (FWHM) of the drift time distribution, which reflects the conformation flexibility of the measured ion,^41–43^ we also observed that the scaffold flexibility was generally reduced after the cyclization process. Finally, not only did the resulting CCS values of scaffolds **3**–**6** indicate more compact sizes of the cyclized scaffolds, but also numerical values provide essential information for constructing structural models in the future.

**Figure 3:**
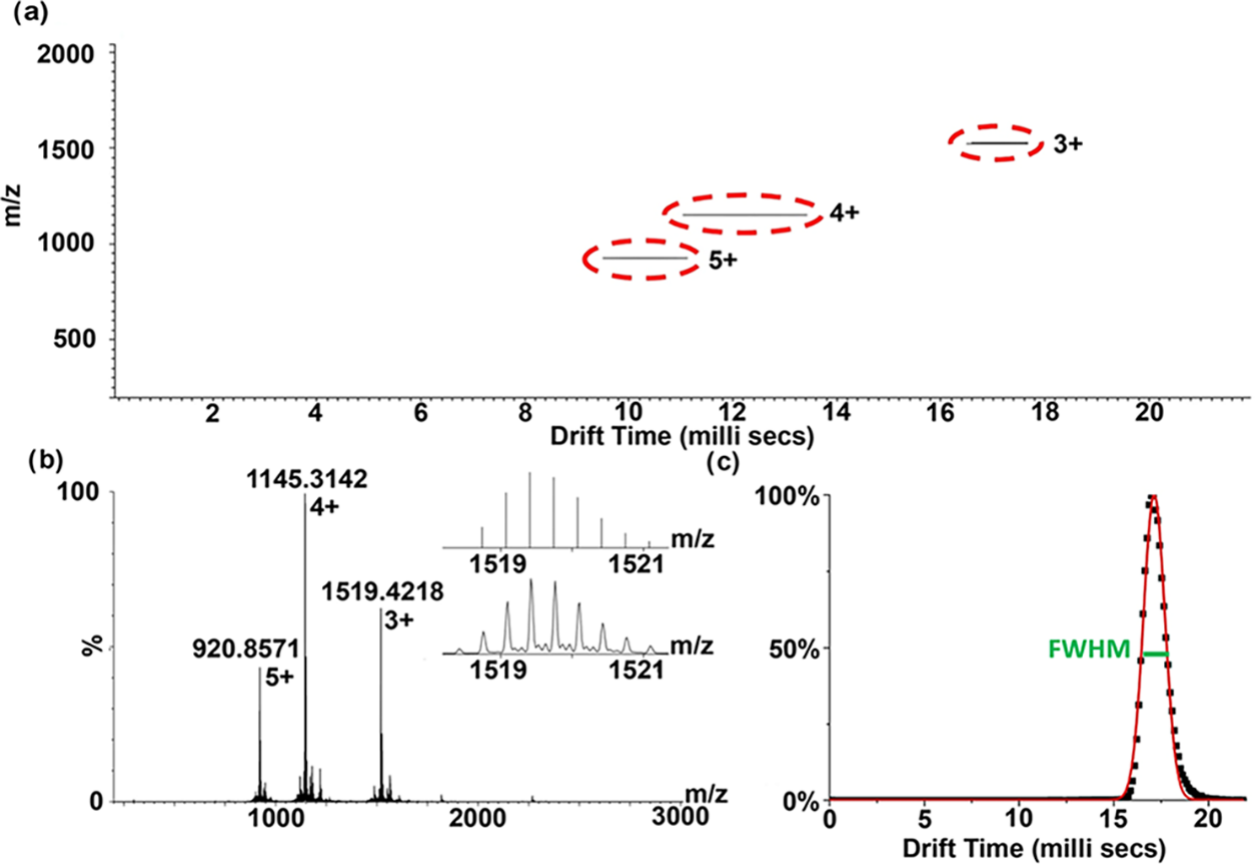
Representative ion mobility spectrometry analysis of cyclic scaffold **6**. (a) TWIM-MS spectrum; (b) mass spectrum of **6**: calculated (inset top) and observed (inset bottom) isotope patterns of [M + 3Na]^3+^; (c) drift time distribution of [M + 3Na]^3+^ with the full width at half-maximum (FWHM) indicated.

**Table 2:** Ion Mobility Spectrometry Parameters for Scaffolds **3–6**

	drift time (ms) and [FWHM^a^] (ms)			
scaffold style	3+ ion	4+ ion	5+ ion	CCS^b^ (Å^2^)
linear (Pro_6_)_4_ **3**	13.67 [1.35]	12.68 [1.95]	10.36 [1.65]	827.53 ± 107.24
linear (Pro_6_)_5_ **4**	17.09 [1.36]	15.33 [1.95]	13.67 [2.33]	972.94 ± 142.62
cyclic (Pro_6_)_4_ **5**	13.34 [1.26]	10.03 [1.53]	7.72 [0.61]	720.20 ± 36.58
cyclic (Pro_6_)_5_ **6**	17.09 [1.33]	11.91 [2.44]	10.25 [1.07]	849.45 ± 57.82

a Full width at half-maxima as an indicator of structural flexibility.

b Collision cross section.

### Glycan Ligand Installation to Cyclic Scaffolds

After characterizing the PP4M and PP5M scaffolds, the next step was to install the Stx B ligand onto the scaffolds. To test whether the cyclic scaffolds were large enough to afford strong multivalent binding with the Stx B pentamer, we designed two linkers of different lengths to connect the Gb3 glycan to the scaffold. The Gb3 installation began by removing the Cbz-protecting groups on **5** and **6** through hydrogenolysis (Scheme 2). The resulting amine groups were coupled to 4-pentynoic acid or an extended alkynyl acid **15** to install alkyne groups at the first position of every Pro_6_ helix of cyclic scaffolds **7**–**10**. Finally, conjugation of multiple Gb3-N_3_ ligands was achieved via the CuAAC reaction to yield Gb3-cyclic scaffolds **11**–**14**. The CD spectra of glycoconjugates **11–14** measured in water (see Supporting Information) with typical PPII characteristics indicated that Gb3 ligand conjugation did not alter the peptide conformation.

**Scheme 2:**
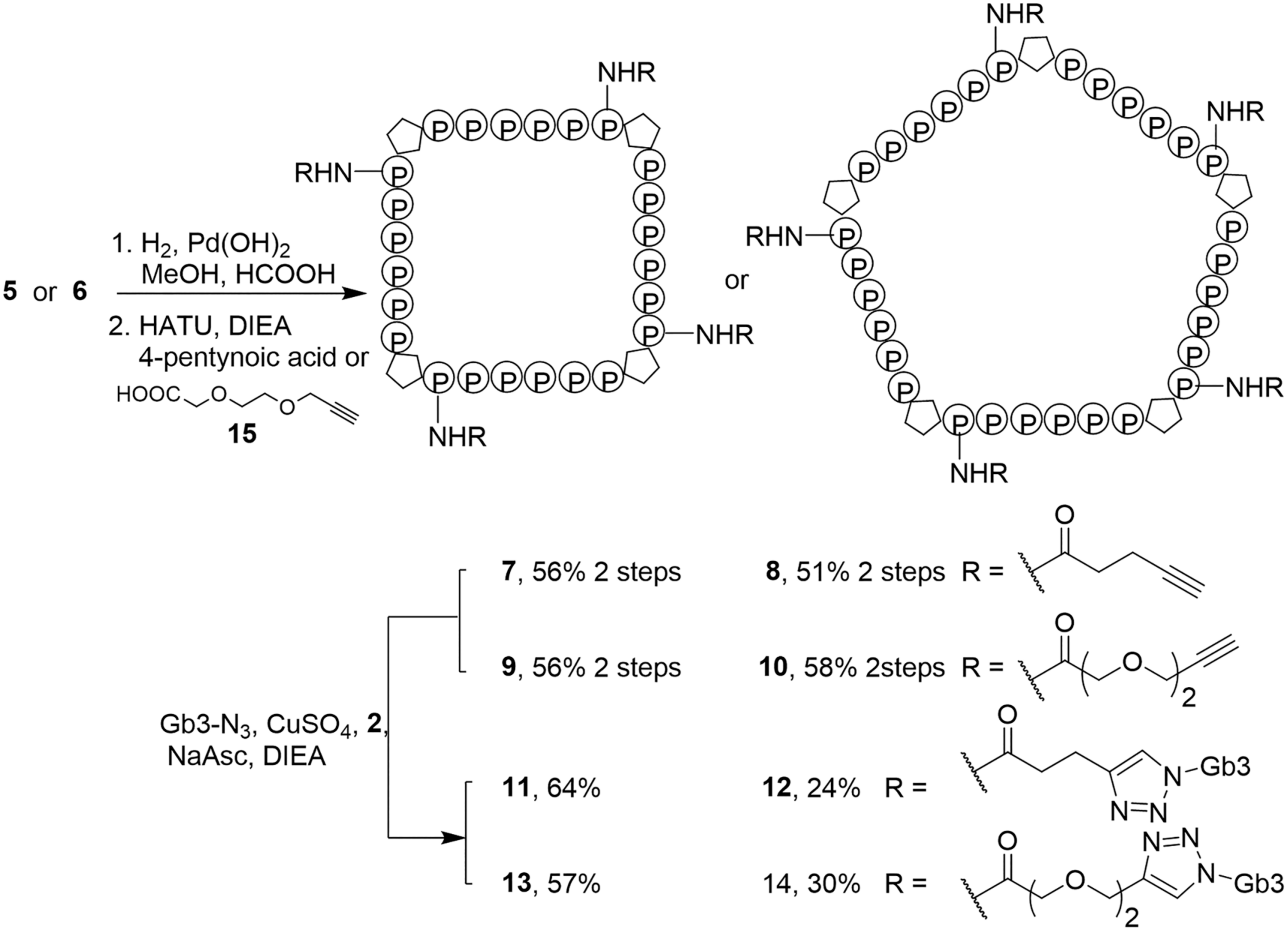
Conjugation of Gb3 Ligands to Obtain Gb3 Conjugates **11**–**14**

### Surface Plasmon Resonance Binding Analysis

After preparing the Gb3 conjugates, we tested their binding to Stx1B through surface plasmon resonance (SPR). To prevent the pseudo multivalency of ligand presentation on the SPR surface, we immobilized Stx1B on the CM5 sensor chip through amide formation. We tested glycoconjugates **11**–**14**, as well as monomeric Gb3-N_3_, at various concentrations, and the resulting sensorgrams (Figure 4 and Supporting Information) were fitted to a 1:1 binding model to acquire their binding parameters for Stx1B (Table 3). The results indicated the importance of multivalent binding, as monomeric Gb3-N_3_ binding was too weak to be measured by SPR. This was not surprising, as the dissociation constant (*K*_D_) of Gb3 trisaccharide to Stx1B was reported to be around 10^–3^ M by isothermal titration calorimetry (ITC) measurements.^44^ In contrast to monomeric Gb3 ligands, multivalent Gb3 on glyconjugates **11**–**14** showed significantly stronger avidity (Table 3). The tetra-helix conjugates **11** and **13** typically have *K*_D_ values of around 1000 nM, whereas the penta-helix counterparts **12** and **14** are about 20-fold stronger. The two types of linkers connecting Gb3 ligands to cyclic scaffolds could have two effects on multivalent binding. First, it is necessary to have a linker that allows the ligand to reach the protein binding site, as well as enabling the ligand to be in an appropriate orientation for protein binding. Second, an excessively long linker may not only result in an entropy penalty upon binding but may also provide too much freedom for the ligands, impairing the precision of positioning controlled by the cyclic scaffolds. For the linker lengths tested, the *K*_D_ comparison between the two linkers (**12** vs **14**) showed no significant difference, suggesting that the designed ligand positions already engaged in strong multivalent interactions such that the extension of the linker did not provide practical aid in binding.

**Figure 4:**
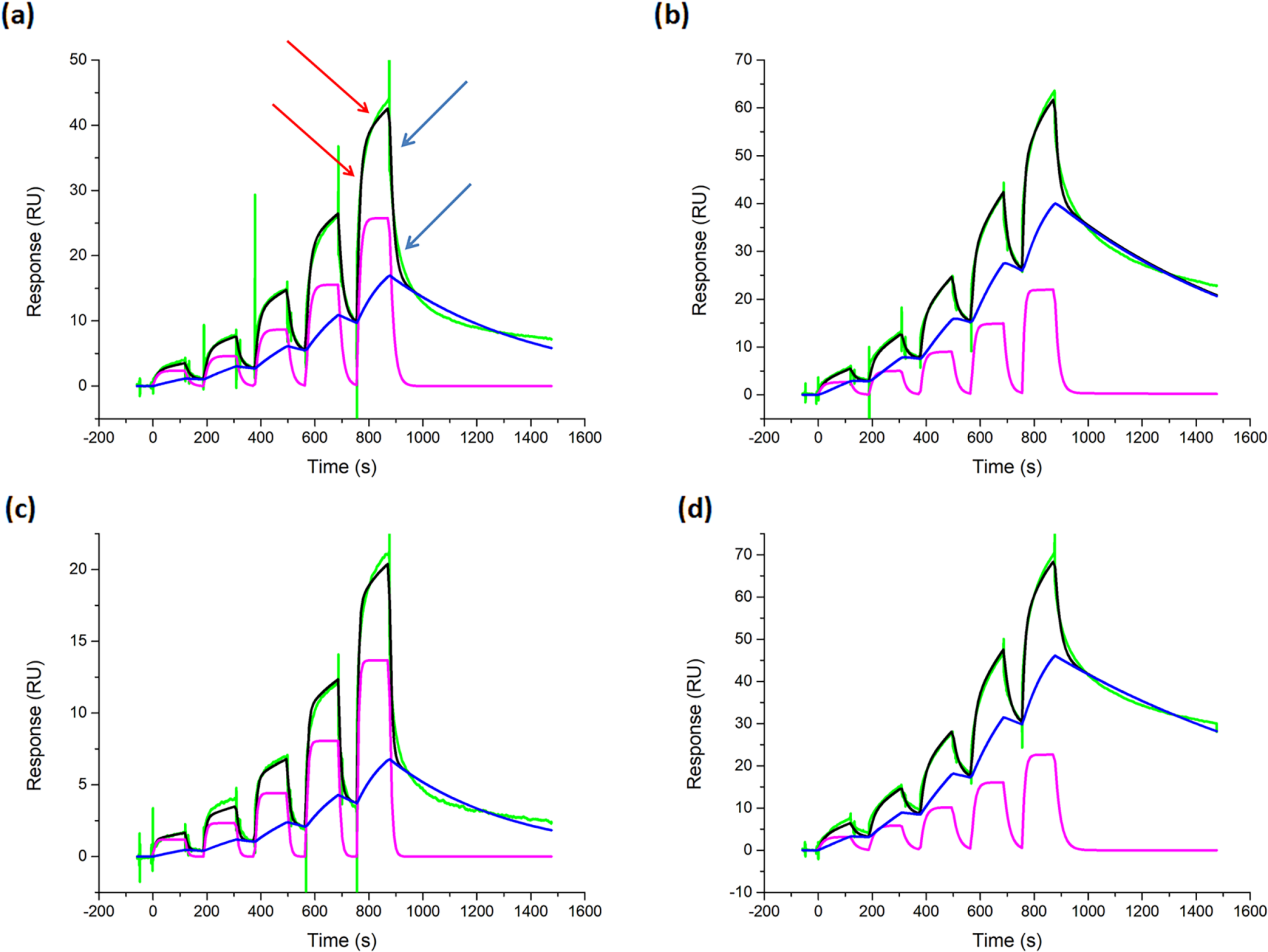
Surface plasmon resonance sensorgrams of Gb3 conjugates **11** (a), **12** (b), **13** (c), and **14** (d). Single-cycle kinetic measurements were performed at 5 concentrations: 125, 250, 500, 1000, and 2000 nM for **11** and **13**; and 31.25, 62.5, 125, 250, and 500 nM for **12** and **14.** The sensorgram curves are shown in green, the two stages of binding events are indicated by red arrows, and the dissociation events are indicated by blue arrows. The heterobinding model fitting curves are shown in black, and the separated curves for the fast and slow binding modes are shown in magenta and blue, respectively.

**Table 3:** SPR Parameters of Conjugate **11**–**14** Binding to Stx1B Fitted with a 1:1 Binding Model

ligand style	*k*_a_ (10^3^/M s)	*k*_d_ (10^–3^/s)	*K*_D_ (nM)	*R*_max_ (RU)	Chi^2^
mono Gb3-N_3_	inactive	inactive	inactive		
short Gb3_4_ **11**	3.38 ± 0.33	2.67 ± 0.51	802 ± 208	39	4.92
short Gb3_5_ **12**	18.4 ± 0.01	1.30 ± 0.04	70.6 ± 2.2	58	3.37
long Gb3_4_ **13**	2.95 ± 0.11	3.37 ± 0.32	1140 ± 160	24	2.33
long Gb3_5_ **14**	22.3 ± 0.66	1.14 ± 0.18	52 ± 6	61	3.94

Interestingly, a detailed examination of the SPR sensorgrams revealed that some association and dissociation curves had roughly two separate stages, as indicated in Figure 4a. Since these stages can represent distinct binding modes and also a heterobinding model in the Biacore Evaluation Software better fits our Stx1B binding events by one analyte binding to two distinct binding sites, we further attempted to fit the sensorgram with this heterobinding model. This process provided two sets of interactions that could be assigned as fast and slow bindings and dissociation to give separated curves (magenta and blue curves in Figure 4, respectively). To evaluate how the heterobinding model fits the measured sensorgrams, we examined the Chi^2^ value of the fitting results (Table 4). Given that Chi^2^ within 10% of the *R*_max_ is often accepted as a good fit, the relatively small Chi^2^ values observed for every tested glycoconjugate **11**–**14** suggested that the heterobinding model was suitable for analyzing the binding events of Stx1B. Therefore, for each of the glycoconjugates, the parameters for both binding modes are listed separately in Table 4 and denoted as “fast” and “slow” binding. The results from this favorable heterobinding model indicated that the *K*_D_ values for penta-Gb3 conjugates **12** and **14** are about 10-fold stronger than those for tetra-Gb3 conjugates **11** and **13** for both fast and slow binding modes. Moreover, the two binding modes contribute differently to the tetra-Gb3 and penta-Gb3-binding events. As indicated separately in the sensorgrams (Figure 4), the major tetra-Gb3 conjugate **11** binding to Stx1B is mainly provided by fast binding (magenta curve), whereas slow binding (cyan curve) is more important for penta-Gb3 conjugate **13**. This binding mode preference can be quantified by the ratio between the *R*_max_, the projected maximum intensity, of the slow and fast binding modes (Table 4).

**Table 4:** SPR Parameters of Conjugates **11**–**14** Binding to Stx1B Fitted with a Heterobinding Model

	slow binding				fast binding					
ligand style	*k*_a_ (10^3^/M s)	*k*_d_ (10^–3^/s)	*K*_D_ (nM)	*R*_max_ (RU)	*k*_a_ (10^4^/M s)	*k*_d_ (10^–2^/s)	*K*_D_ (nM)	*R*_max_ (RU)	slow/fast binding ratio^a^	Chi^2^
short Gb3_4_ **11**	3.09 ± 0.51	1.78 ± 0.01	583 ± 106	28	1.70 ± 0.13	6.63 ± 0.18	3910 ± 200	76	0.36	1.74
short Gb3_5_ **12**	17.8 ± 0.6	1.12 ± 0.05	63.0 ± 0.8	53	19.3 ± 8.3	8.97 ± 2.83	482 ± 74	43	1.23	1.30
long Gb3_4_ **13**	3.53 ± 0.43	2.28 ± 0.20	647 ± 48	11	2.32 ± 0.48	12.8 ± 0.1	5600 ± 990	52	0.21	0.31
long Gb3_5_ **14**	16.0 ± 0.6	0.85 ± 0.03	53.1 ± 0.1	58	13.2 ± 1.1	5.37 ± 0.43	410 ± 64	40	1.45	1.40

a The slow and fast binding ratios are presented as *R*_max, slow_ to *R*_max, fast_.

To explain how the alternative Gb3 ligand arrangements lead to opposite preferences in the stronger (lower *K*_D_) slow binding mode or the weaker (higher *K*_D_) fast binding mode, it is necessary to consider the different binding sites within an Stx B subunit. Crystallographic investigations have identified three Gb3-binding sites, known as site 1, site 2, and site 3 (Figure 5), on Stx1B.^13^ Later, by measuring the *K*_D_ of multivalent Gb3 conjugates to various site-directed Stx1B mutant strains, researchers revealed the binding preferences for the three sites. Site 3, close to the core of the Stx1B pentamer, has the weakest binding avidity, whereas Site 2, at the edge of the pentamer, has the strongest.^45^ Based on the Gb3-complexed Stx1B structure,^13^ the distance between site 3 of the neighboring B subunits is around 1.2 nm, whereas that of site 2 is about 2.7 nm. We estimated^6,18^ the dimensions of both the PP4M and PP5M scaffolds as polygons with about 2 nm lengths for each constituting helix. When the projected scaffold dimensions were superimposed on the binding sites of the Stx1B pentamer, we found that the PP5M scaffold fit the five stronger Gb3-binding sites 2 better due to its size and ligand arrangement (Figure 5) and likely contributed more to the stronger “slow” binding mode to give higher slow/fast binding ratios of 1.23 and 1.45 for **12** and **14**, respectively (Table 4). In contrast, PP4M scaffolds **11** and **13** were smaller and not capable of reaching as many sites 2 as the PP5M conjugates, resulting in low slow/fast binding ratios of 0.36 and 0.21, respectively. The current heterobinding model separates two binding types (namely, slow and fast), and these results do not rule out the possibility of mixed binding among sites 1, 2, and 3. Such a possibility would require the development of a more complicated binding algorithm to process the SPR sensorgrams for evaluation. Nevertheless, the relative strengths of the slow and fast binding modes are controlled by the designed Gb3 ligand presentation on the scaffold, and this approach allows monitoring of the resultant selectivity.

**Figure 5:**
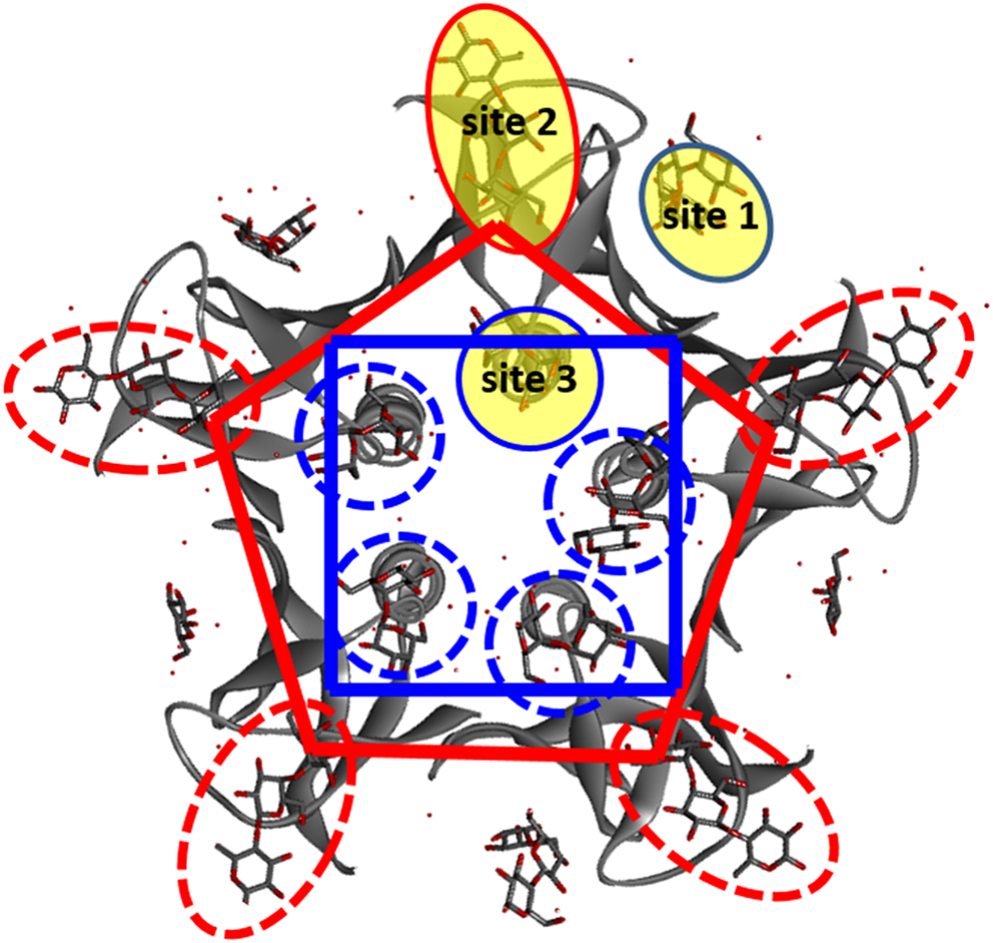
Overlaying the projected dimensions of cyclic scaffolds **5** (blue) and **6** (red) with the Gb3-complexed crystal structure of Stx1B based on ref 13. The binding sites within one subunit are indicated by yellow areas.

Over the past few decades, significant advancements in synthetic methodologies have accelerated progress in the field of carbohydrate synthesis. Despite these achievements, the recognition of glycans and proteins remains a major challenge. A key issue is cross-reactivity, wherein a protein may interact with structurally similar glycans presented on various biomolecules, and conversely, a single glycan structure can also be recognized by different proteins. This complexity in glycan–protein interactions can be addressed by employing glycan patterning to achieve multivalent recognition, thereby enhancing the binding selectivity toward clustered protein receptors.^3^ Such recognition has been exploited in the immune system to sense pathogen-associated molecular patterns and has potential in many therapeutic applications.^46^ Studying clustered membrane receptors presents several challenges, including verifying their existence as clusters and understanding the spatial organization of receptors within these clusters in relation to cellular function. One investigative approach involves modulating receptor distribution by binding them to precisely controlled ligand patterns at the nanoscale.^47^ Therefore, the ability to engineer ligand patterns with high precision on nanoscaffolds not only advances our understanding of receptor clustering but also holds significant potential for modulating receptor organization in nanomedicine applications.

To target multisubunit protein receptors, previous quantitative predictions suggest that an ideal scaffold core matches the receptor size with linkers slightly longer than the separation between the core and receptor.^48^ Using our rigid nanosized cyclic scaffold system, we can fully control the ligand pattern based on the peptide sequence and the order of peptide helix ligation. Compared to the traditional multivalent design using a smaller core with long linkers to reach the dimensions of protein oligomers at several nanometers (e.g., ref 45), our design does not necessarily give a better *K*_D_, as our scaffold allows a smaller degree of freedom. However, due to the lack of freedom for ligands to adapt to nontarget proteins, the selectivity for the target protein can significantly increase. This phenomenon was observed in our previous work for the selective targeting of the DC-SIGN tetramer over the langerin trimer by presenting Man_4_ on a PP4M scaffold. We found that the dendrimer carrying 9 Man_4_ ligands has stronger binding (*K*_D_ = 0.7 nM, likely due to the statistical rebinding effect) than our 4 Man_4_-carrying PP4M conjugate (*K*_D_ = 3.4 nM), but Man_4_-PP4M has about 30-fold better DC-SIGN/langerin selectivity than the Man_4_ dendrimer.^6^ Other groups have also separately exploited rigid scaffolds in combination with branch-clustered ligands to target DC-SIGN^49^ or langerin.^50^ Both groups reported that the receptor-matching separation of ligands on the scaffold provides good binding through the chelation effect, but the local cluster of excessive ligands further boosts avidity through the statistical rebinding effect. However, excessive ligands may also lead to unwanted binding, including the clustering of clustered receptors,^45,49,51^ and it is unclear whether the selectivity among receptors is affected by such a design. Our present work raises the challenge to another level to selectively bind heterosites on the same protein, and therefore, excessive ligands from local ligand clusters were avoided in our design to simplify the investigation. We observed that changes in the ligand patterns led to different propensities of the binding modes that were consistent with the binding site affinity. These results demonstrated the capability of the PPnM scaffolds for selective multivalent carbohydrate–protein interactions.

In addition to the cyclic scaffold design and synthesis, the combination of kinetic analysis from SPR and the heterobinding model allows us to separate two types of binding modes. With information on the individual binding site affinity, it is possible to evaluate the multiple ligand arrangement and the corresponding contribution to each binding mode. This analysis allows us to conveniently observe binding mode alterations in response to ligand arrangement modifications. Without this approach, a series of proteins with mutations at various binding sites would be required to test each individual ligand pattern analog,^45^ which would require a significant amount of time and effort. Moreover, even if such an analysis is performed, it would not provide the relative contributions of the different binding modes. Therefore, using kinetic SPR analysis with heterobinding model fitting can provide valuable information to complement traditional mutant analysis. Even when this approach is used alone, such an analysis provides useful information.

## Conclusions

In conclusion, we have developed an unprecedented pentapolyproline helix cyclic scaffold and characterized it using CD spectroscopy and TWIM-MS to estimate its dimensions. These efforts expand the arsenal of fully adjustable cyclized polypropylene helical nanoscaffold systems for the creation of more precise ligand patterns toward the desired binding sites at the nanoscale. We found that the Gb3 ligand presentation in different patterns favors alternative binding modes to Stx1B, indicating that the binding site selectivity in the same protein oligomer can be manipulated by the ligand spatial arrangement on the PPnM scaffold system. This precision in ligand positioning to match stronger binding sites can also enhance the selectivity against other cross-reactive nontarget proteins. Our SPR kinetic binding analysis of multivalent ligands to the complicated protein target allowed the separation of binding modes to evaluate the fitness of the ligand pattern to match the arrangements of nonequivalent binding sites. The combination of these tools in synthesis and analysis not only allows the manipulation of essential multivalent selectivity for Shiga toxin to improve its therapeutic applications but also provides an essential toolkit for extended investigations of other complicated multivalent carbohydrate–protein binding systems.

## Supplementary Material



## Notes

Details of peptide characterization, additional SPR sensorgrams, and CD spectra. (PDF)

## Abbreviations

CCS: collision cross section
CD: circular dichroism
DC-SIGN: dendritic cell-specific intercellular adhesion molecule-3-grabbing nonintegrin
FWHM: full width at half-maximum
Gb3: globotriose
HUS: hemolytic-uremic syndrome
ITC: isothermal titration calorimetry
PPII: polyproline helix type 2
PPnM: polyproline oligo-helix macrocycle
SPR: surface plasmon resonance
Stx: Shiga toxin
STEC: Shiga toxin-producing *E. coli*
TWIM-MS: traveling wave ion mobility mass spectrometry
