# Supplementary material for: Dissecting Multivalent Carbohydrate Binding through Controlled Ligand Patterns on Cyclic Nanoscaffolds
Source: Biomacromolecules. 2026 Apr 24;27(5):3199–209. doi: 10.1021/acs.biomac.5c02630 (PMC13169358; doi:10.1021/acs.biomac.5c02630)
Supplement: Supplementary file 1 [file bm5c02630_si_001.pdf]

Dissecting multivalent carbohydrate binding through controlled ligand pattern on cyclic nanoscaffolds

Supporting Information

Xin-Yu Wang,[1] Zong-You Lee,[1] Meng-Che Li,[1] Masayuki Hashimoto,[2] Kwun-Yung Cheung,[3] Yi-Tsu Chan,[3] Wei-Chieh Cheng,[4] Sheng-Kai Wang*,[1]

Affiliation

1 Department of Chemistry, National Tsing Hua University, Hsinchu 300044, Taiwan

2 Institute of Molecular Medicine, National Cheng Kung University, Tainan 701401, Taiwan

3 Department of Chemistry, National Taiwan University, Taipei 106319, Taiwan

4 Genomics Research Center, Academia Sinica, Taipei 115024, Taiwan

* **Corresponding Author**

*Email: skwang@mx.nthu.edu.tw

Table of Contents

Analytical data of peptide scaffolds S2

CD spectra of peptide scaffolds S15

IMS-MS data of peptide scaffolds S16

SPR sensorgrams S19

Analytical data of peptide scaffolds

Peptide **1**

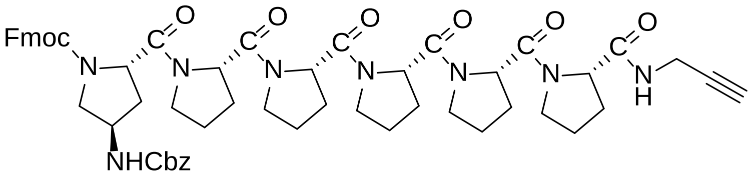

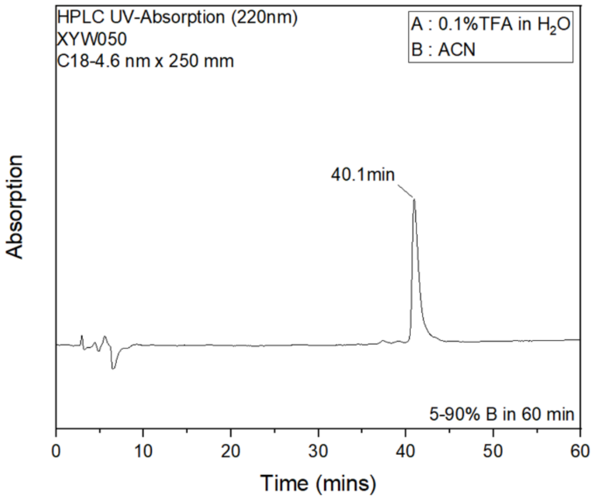

Figure S1. HPLC chromatogram of **1**

Yield: 146.9 mg, 72%

Analytical HPLC: 5% to 90% B with A over 60 mins, 0.5 mL/min；t_R = 40.1 min.

MS (MALDI): $[M+Na]^+$ calcd. For $C_{56}H_{64}N_8NaO_{10}^+$: 1031.463, found: 1031.79

Peptide **3**

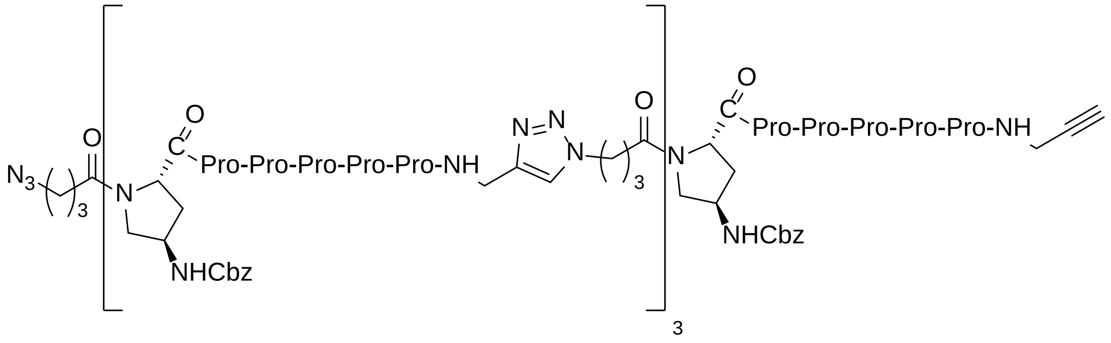

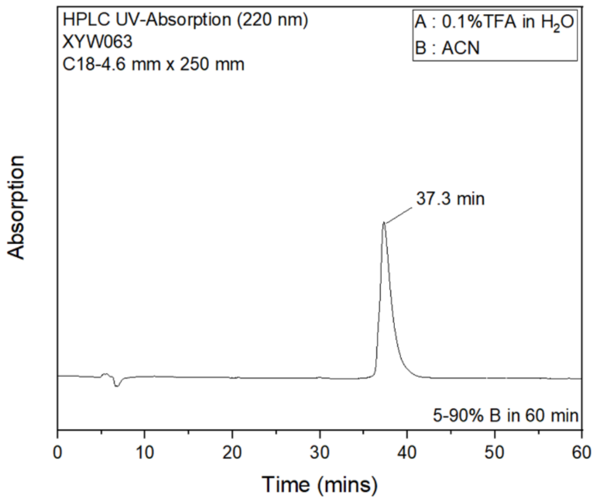

Figure S2. HPLC chromatogram of **3**

Yield: 18.8 mg, 74%

Analytical HPLC: 5% to 90% B with A over 60 mins, 0.5 mL/min ; t_R = 37.3 min.

MS (MALDI): $[M+Na]^+$ calcd. For $C_{180}H_{236}N_{44}NaO_{36}{}^+$: 3612.788, found: 3614.998

Peptide **4**

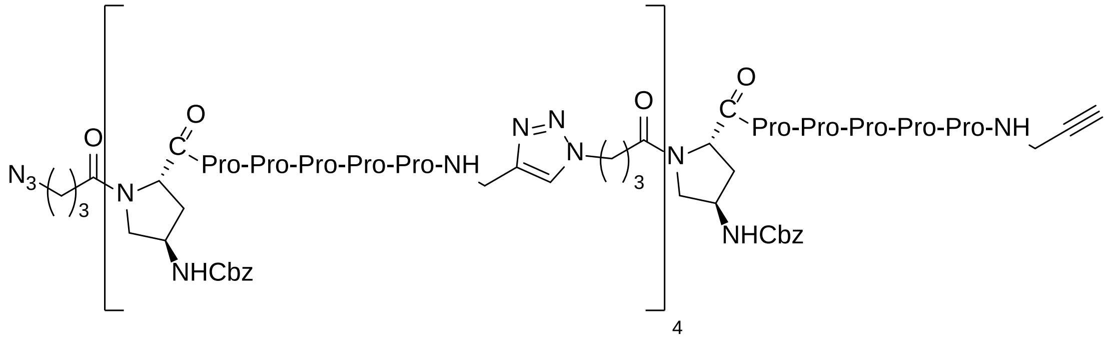

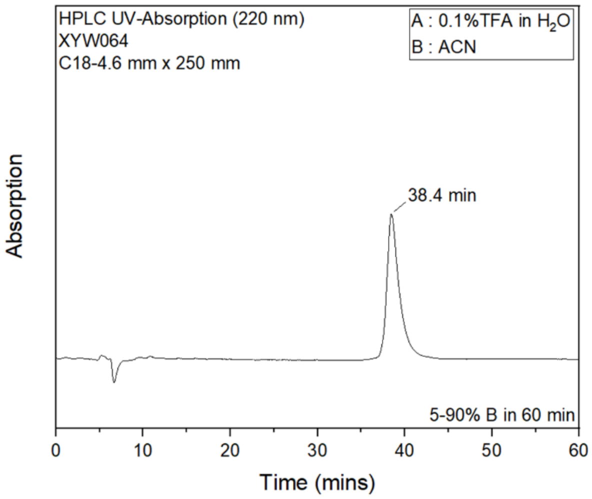

Figure S3. HPLC chromatogram of **4**

Yield: 46.1 mg, 53%

Analytical HPLC: 5% to 90% B with A over 60 mins, 0.5 mL/min ; t_R = 38.4 min.

MS (MALDI): $[M+Na]^+$ calcd. For $C_{225}H_{295}N_{55}NaO_{45}^+$: 4510.237, found: 4514.568

Peptide **5**

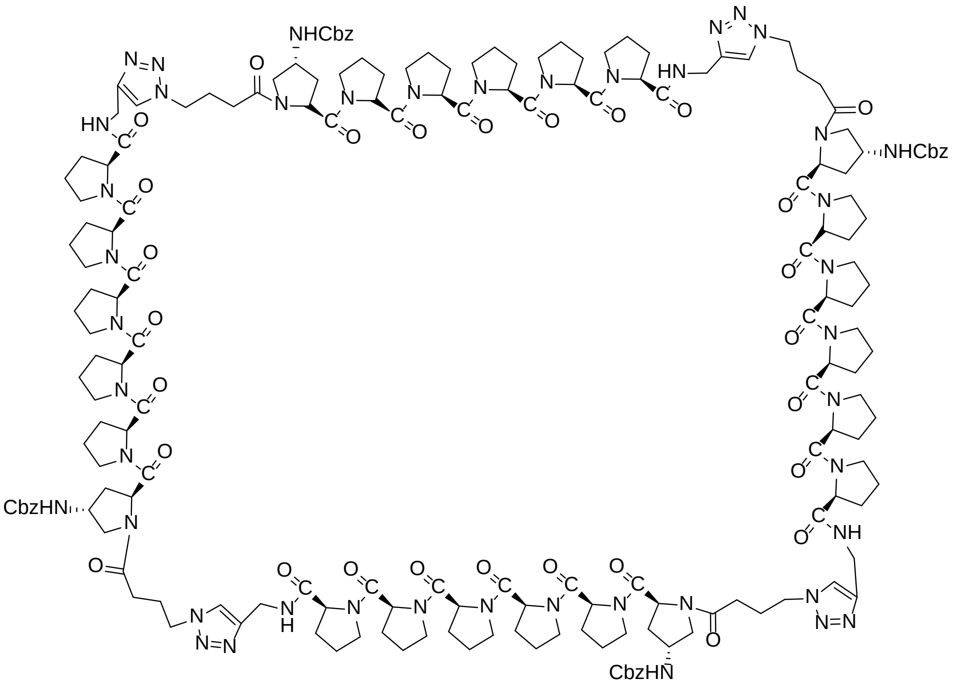

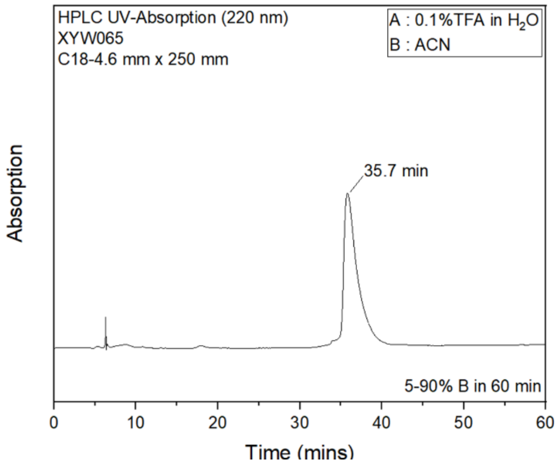

Figure S4. HPLC chromatogram of **5**

Yield: 6.3 mg, 34%

Analytical HPLC: 5% to 90% B with A over 60 mins, 0.5 mL/min；tR = 35.7 min.

MS (MALDI): [M+Na]+ calcd. For $C_{180}H_{236}N_{44}NaO_{36}{}^{+}$: 3612.788, found: 3612.908

Peptide **6**

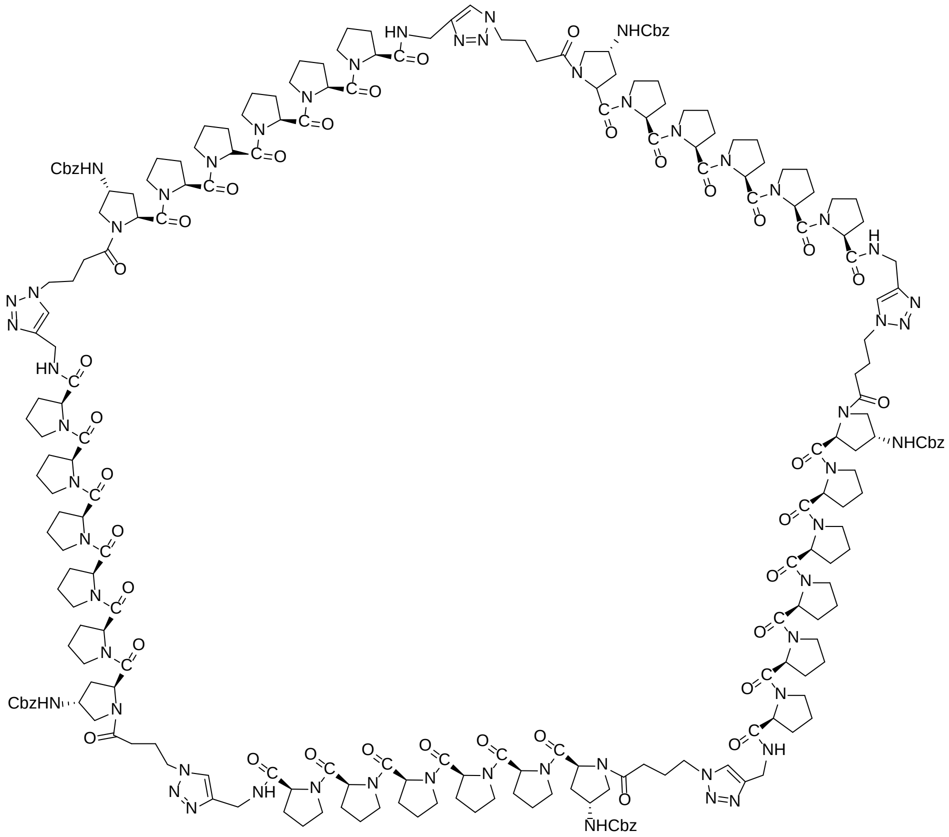

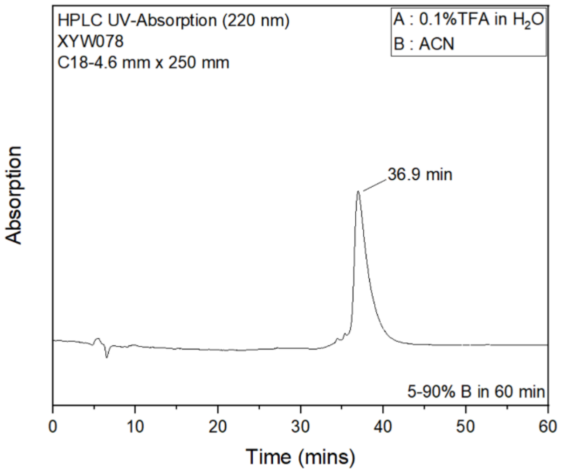

Figure S5. HPLC chromatogram of **6**

Yield: 4.8 mg, 37%

Analytical HPLC: 5% to 90% B with A over 60 mins, 0.5 mL/min；t_R = 36.9 min.

MS (MALDI): $[M+Na]^+$ calcd. For $C_{225}H_{295}N_{55}NaO_{45}{}^+$: 4510.237, found: 4510.549

Peptide **7**

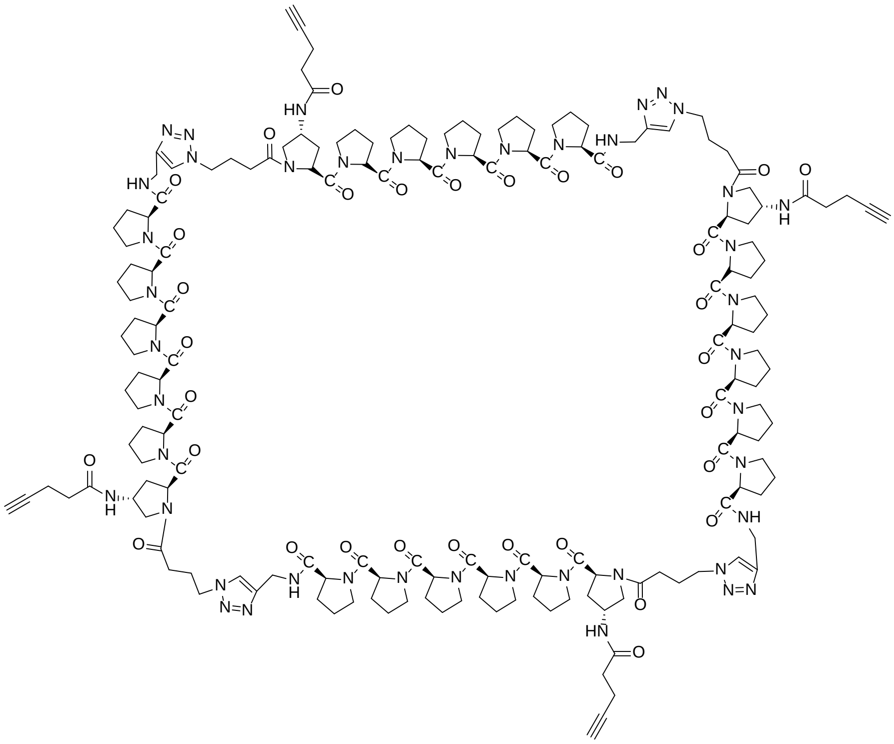

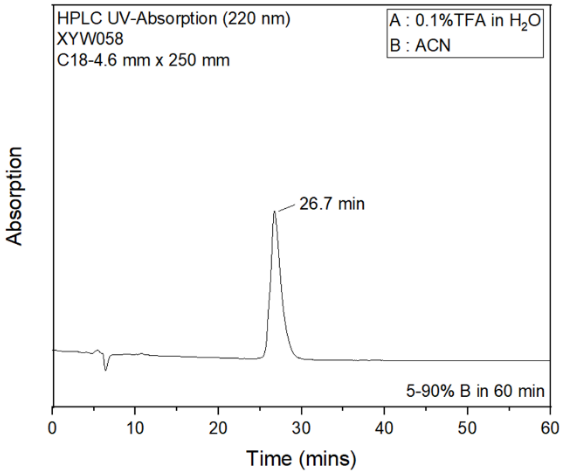

Figure S6. HPLC chromatogram of **7**

Yield: 0.8 mg, 56%

Analytical HPLC: 5% to 90% B with A over 60 mins, 0.5 mL/min；t_R = 26.7 min.

MS (MALDI): $[M+Na]^+$ calcd. For $C_{168}H_{228}N_{44}NaO_{32}^+$: 3396.745, found: 3398.135

Peptide **8**

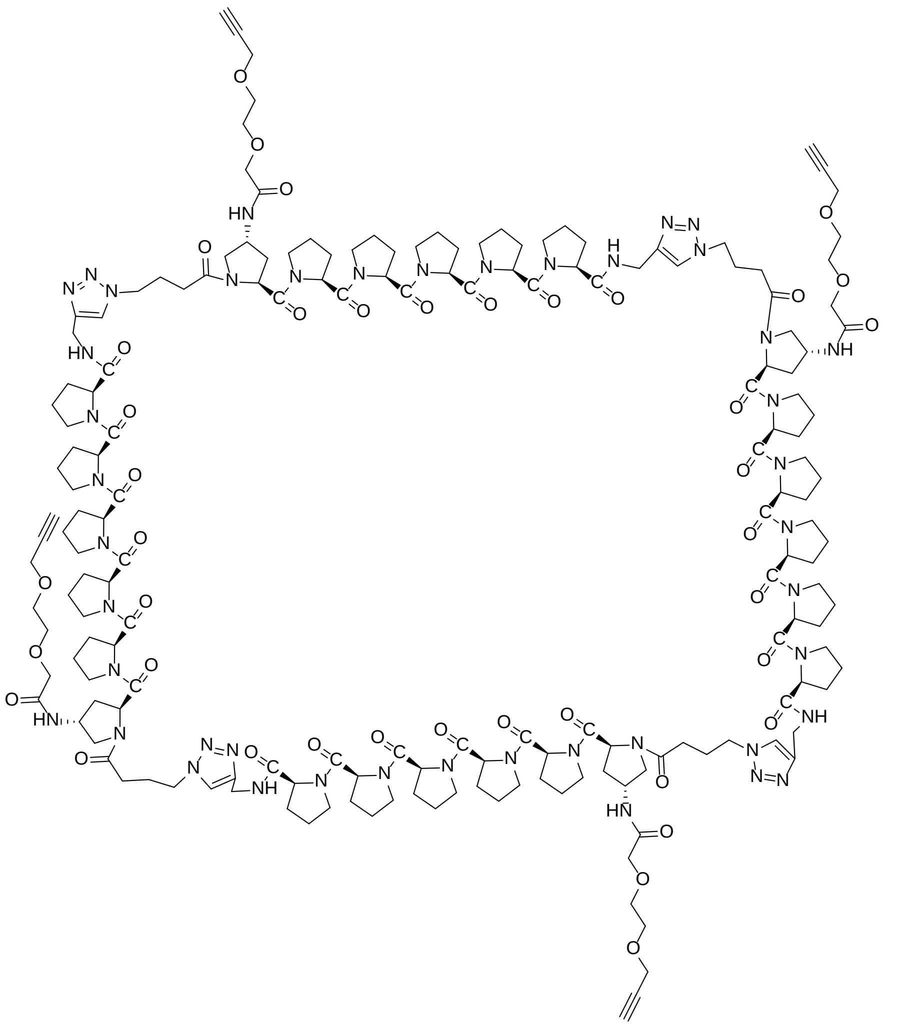

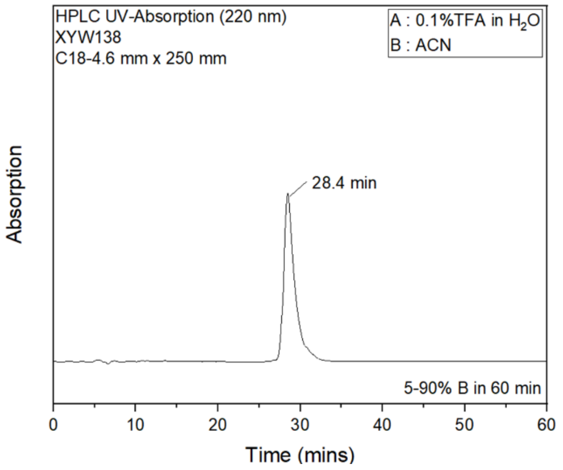

Figure S7. HPLC chromatogram of **8**

Yield: 0.5 mg, 56%

Analytical HPLC: 5% to 90% B with A over 60 mins, 0.5 mL/min；tR = 28.4 min.

MS (MALDI): [M+Na]+ calcd. For $C_{176}H_{244}N_{44}NaO_{40}^{+}$: 3636.830 found: 3637.287

Peptide **9**

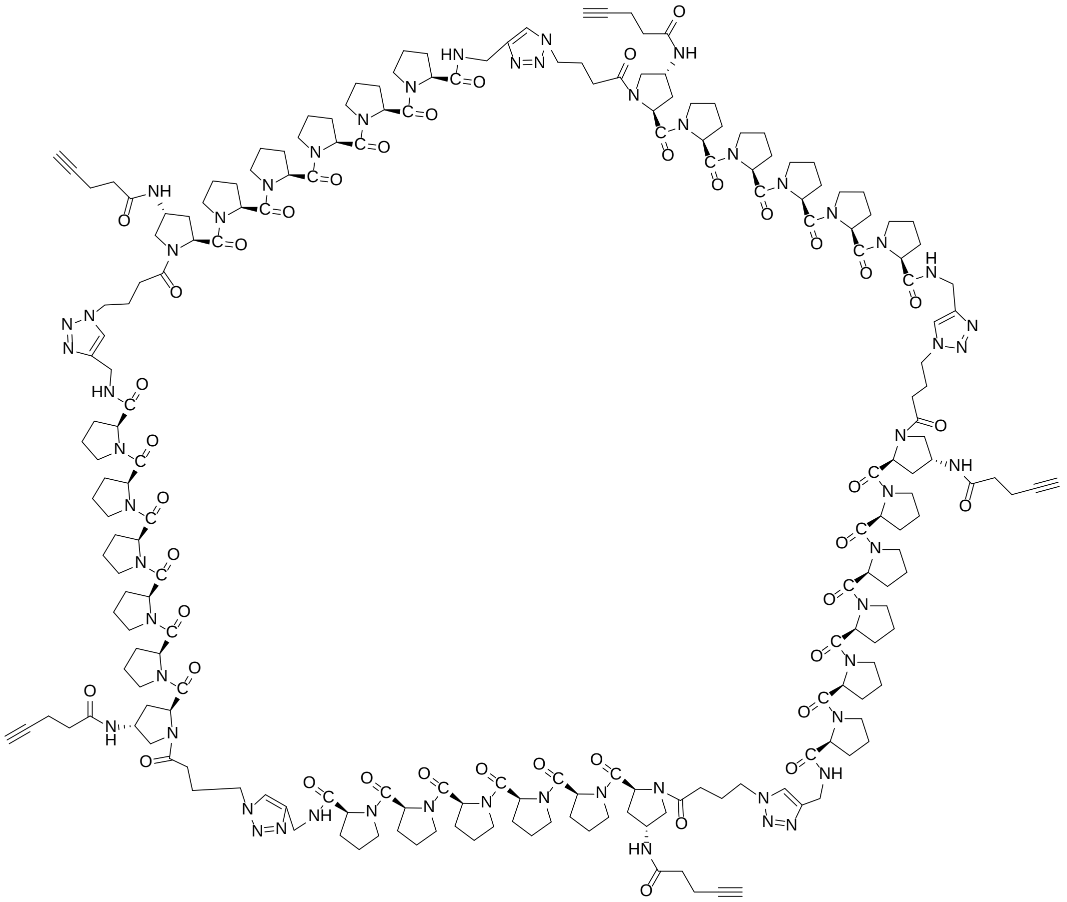

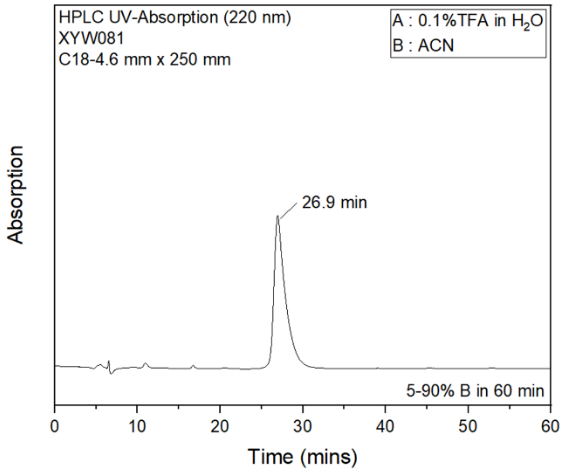

Figure S8. HPLC chromatogram of **9**

Yield: 0.8 mg, 51%

Analytical HPLC: 5% to 90% B with A over 60 mins, 0.5 mL/min；t_R = 26.9 min.

MS (MALDI): $[M+Na]^+$ calcd. For $C_{210}H_{285}N_{55}NaO_{40}^+$: 4240.185, found: 4240.647

Peptide **10**

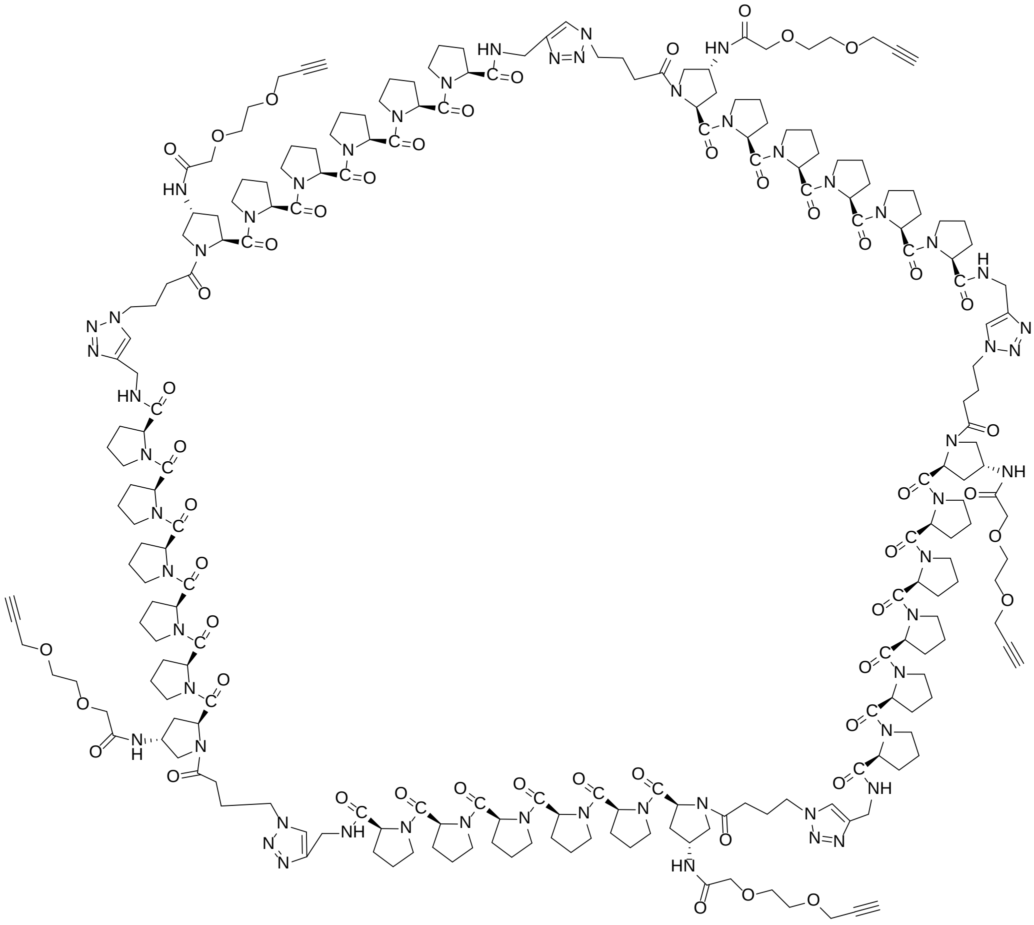

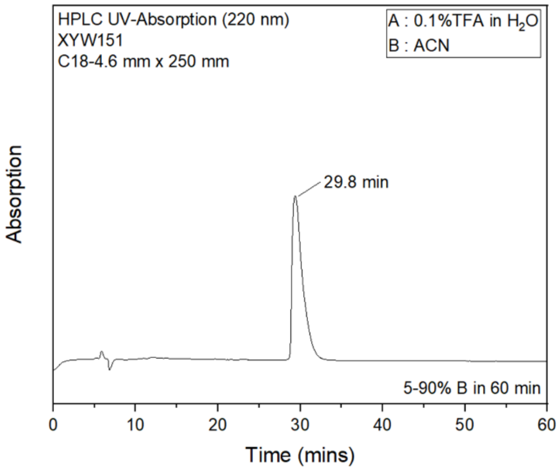

Figure S9. HPLC chromatogram of **10**

Yield: 1.0 mg, 58%

Analytical HPLC: 5% to 90% B with A over 60 mins, 0.5 mL/min ; t_R = 29.8 min.

MS (MALDI): $[M+Na]^+$ calcd. For $C_{220}H_{305}N_{55}NaO_{50}^+$: 4540.290, found:4539.479

Peptide **11**

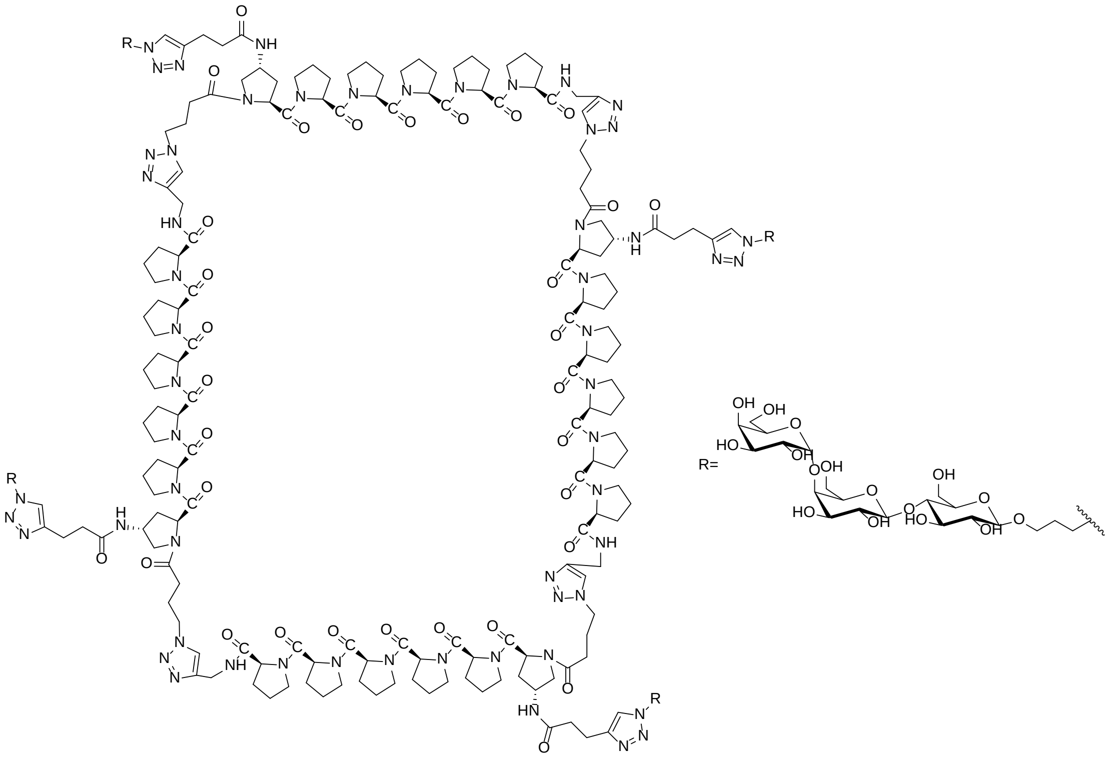

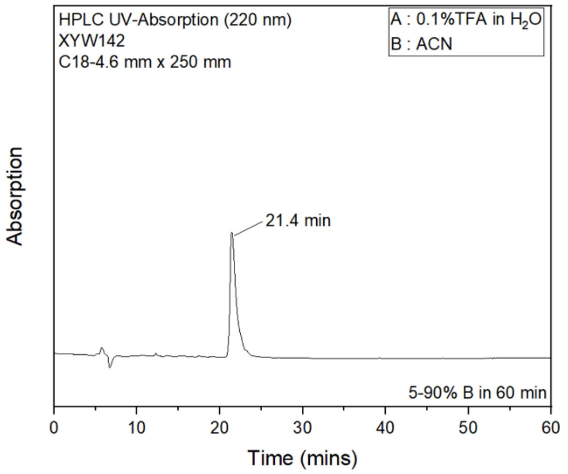

Figure S10. HPLC chromatogram of **11**

Yield: 0.6 mg, 64%

Analytical HPLC: 5% to 90% B with A over 60 mins, 0.5 mL/min ; t_R = 21.4 min.

MS (MALDI): $[M+Na]^+$ calcd. For $C_{252}H_{376}N_{56}NaO_{96}^+$: 5745.615, found: 5744.901

Peptide **12**

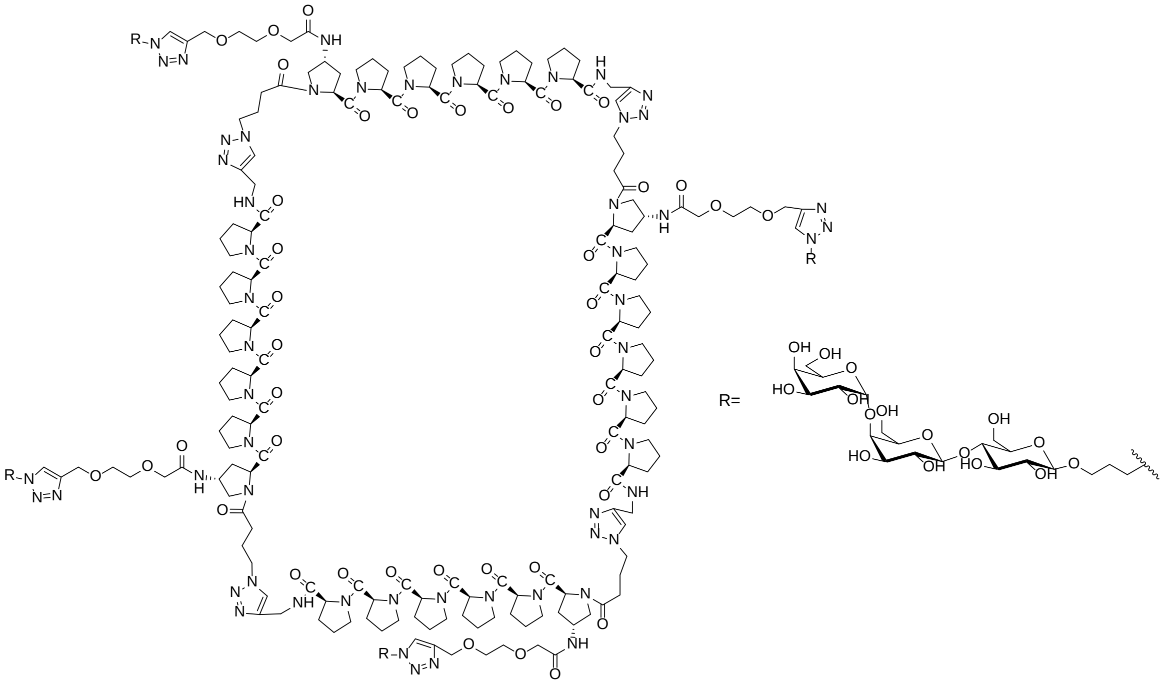

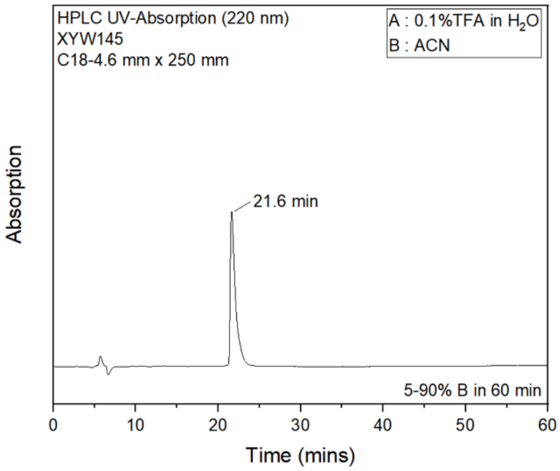

Figure S11. HPLC chromatogram of **12**

Yield: 0.5 mg, 57%

Analytical HPLC: 5% to 90% B with A over 60 mins, 0.5 mL/min；t_R = 21.6 min.

MS (MALDI): $[M+Na]^+$ calcd. For $C_{260}H_{392}N_{56}NaO_{104}^+$: 5985.699, found: 5985.724

Peptide **13**

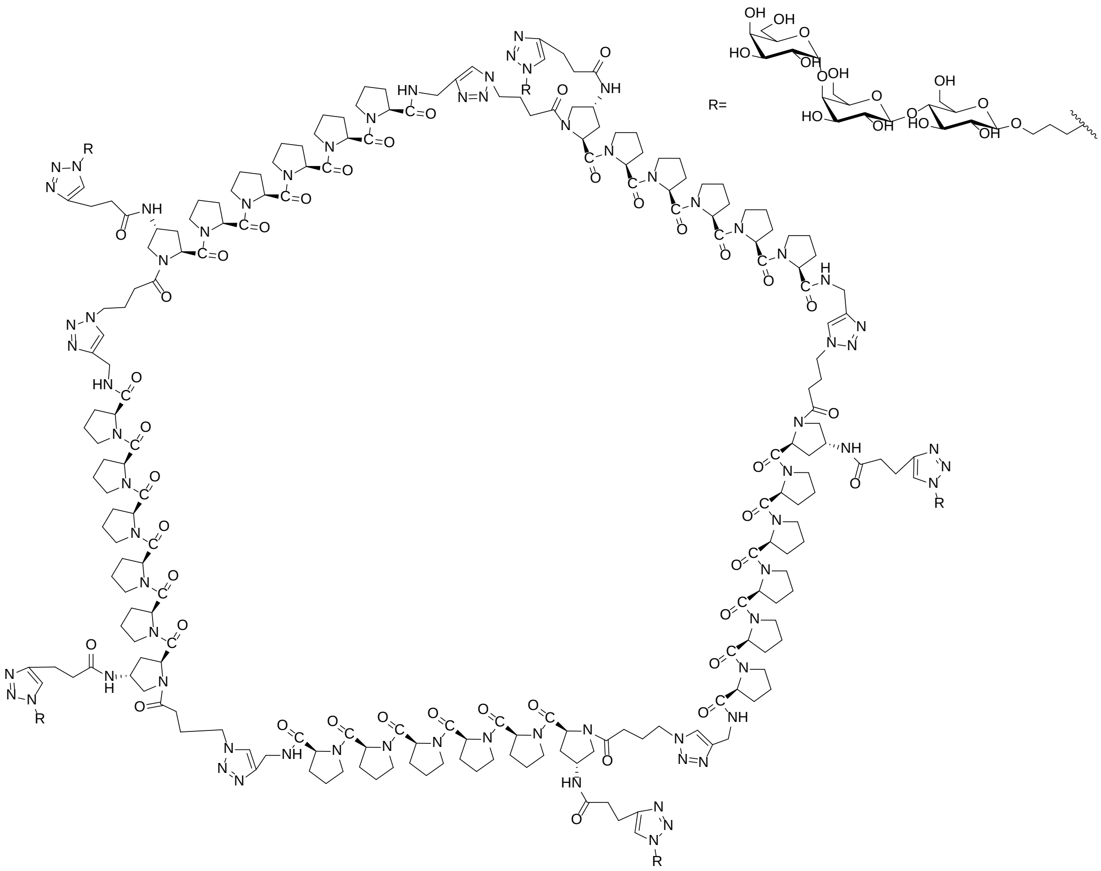

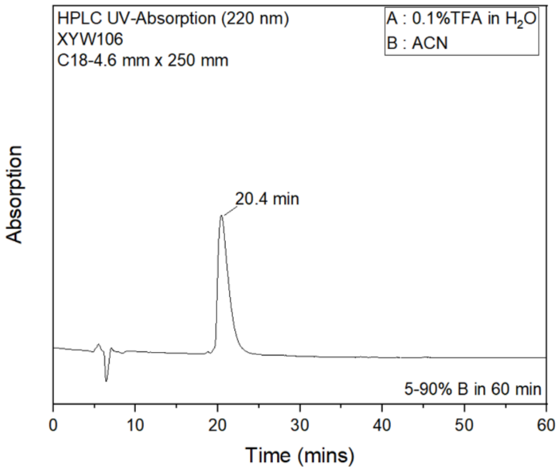

Figure S12. HPLC chromatogram of **13**

Yield: 0.2 mg, 24%

Analytical HPLC: 5% to 90% B with A over 60 mins, 0.5 mL/min ; t_R = 20.4 min.

MS (MALDI): $[M+Na]^+$ calcd. For $C_{315}H_{470}N_{70}NaO_{120}{}^+$: 7176.271, found: 7176.388

Peptide **14**

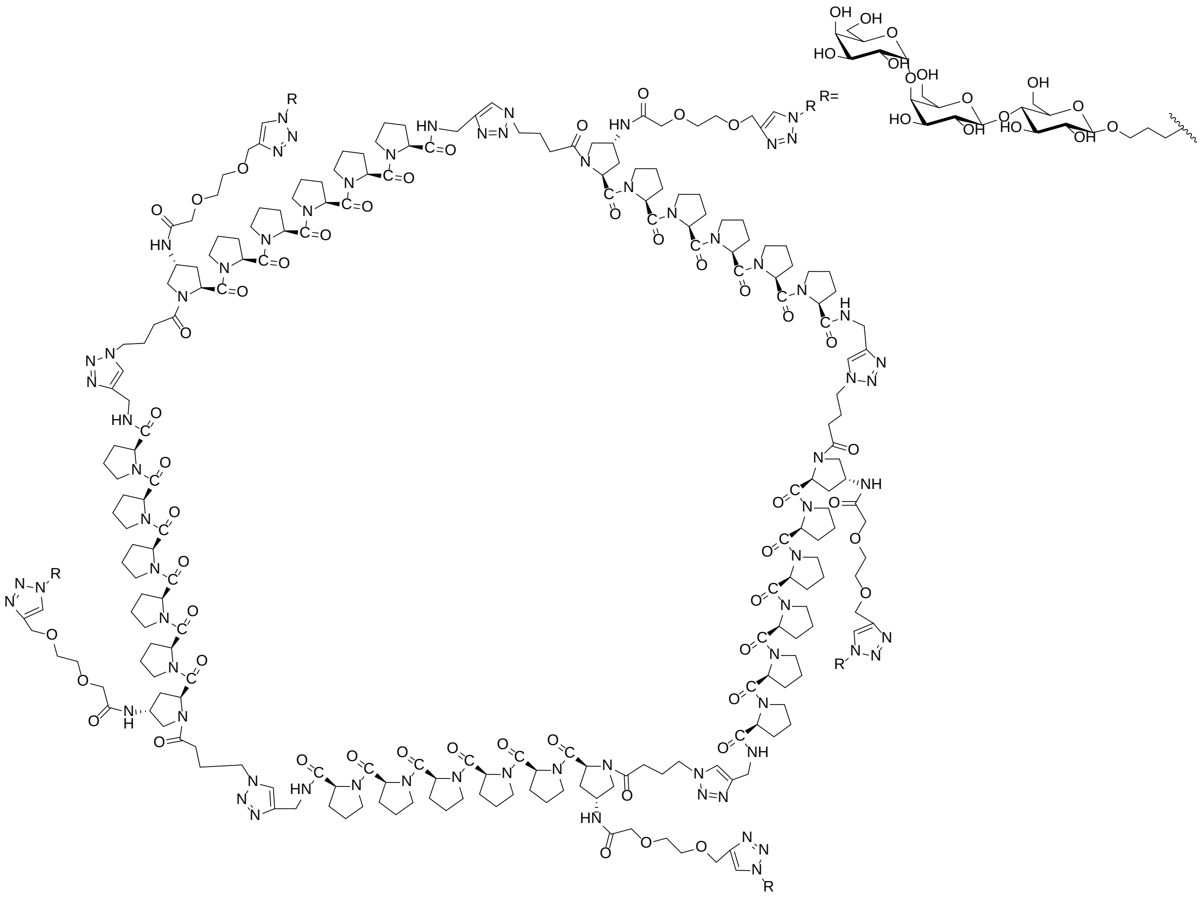

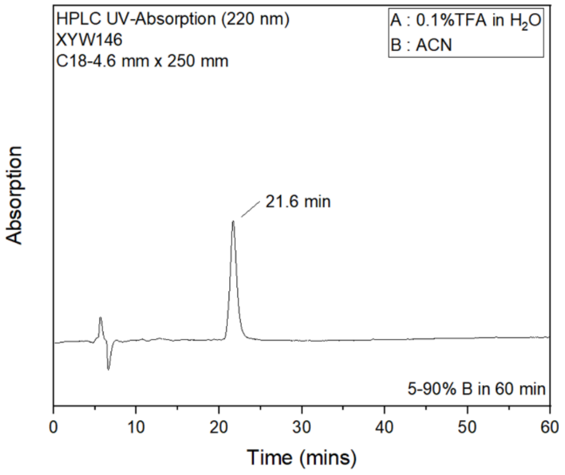

Figure S13. HPLC chromatogram of **14**

Yield: 0.5 mg, 30%

Analytical HPLC: 5% to 90% B with A over 60 mins, 0.5 mL/min ; t_R = 21.6 min.

MS (MALDI): $[M+Na]^+$ calcd. For $C_{325}H_{490}N_{70}NaO_{130}{}^+$: 7476.377, found: 7474.210

CD spectra of peptide scaffolds

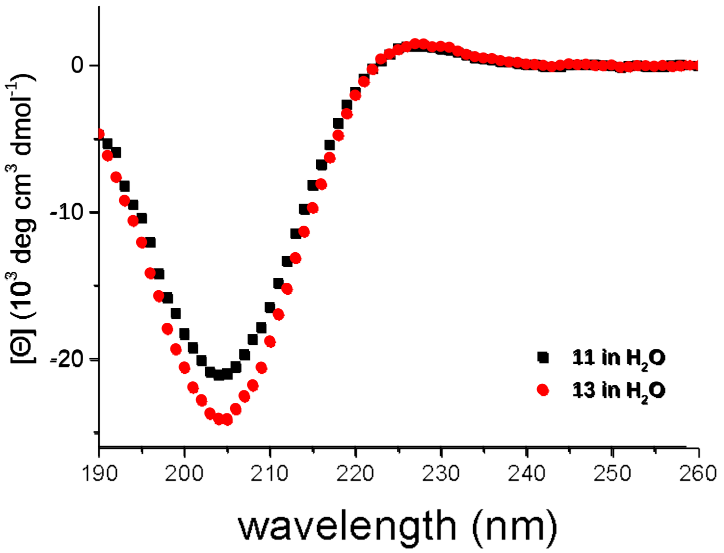

Figure S14. CD spectra of glycopeptide scaffolds **11** and **13** in H_2O

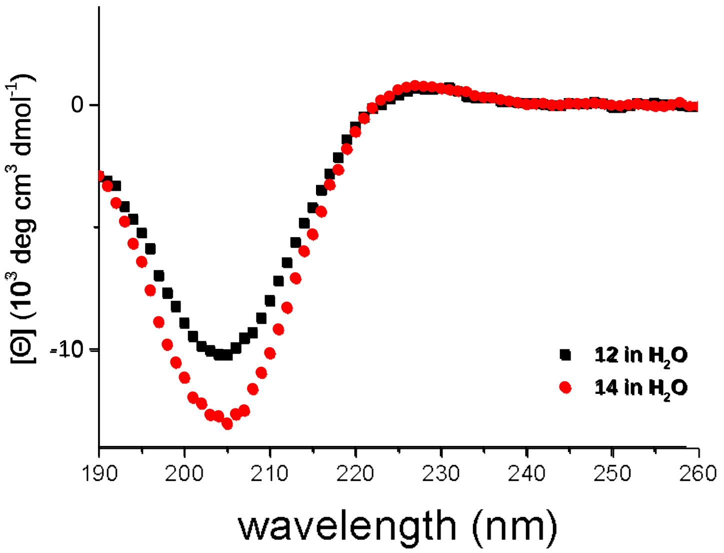

Figure S15. CD spectra of glycopeptide scaffolds **12** and **14** in H_2O

IMS-MS data of peptide scaffolds

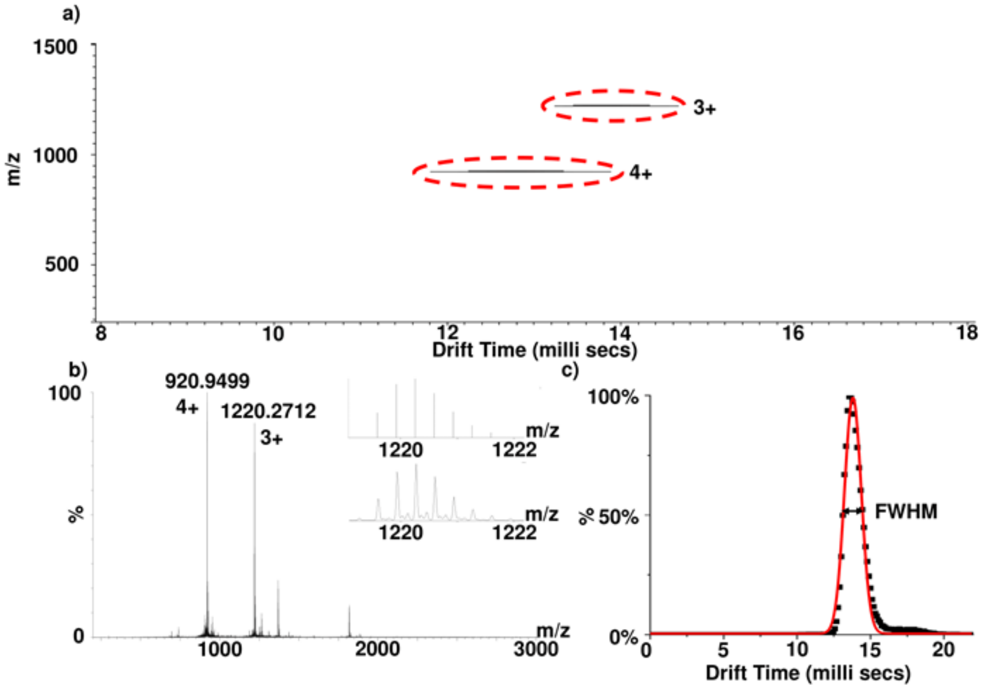

Figure S16. (a) IMS-MS spectrum; (b) isotope pattern and (c) drift-time distribution of [**3** + 3Na]$^{3+}$. The drift-time distribution was fitted by Gaussian normal distribution (red line) from the experimental data (black dots).

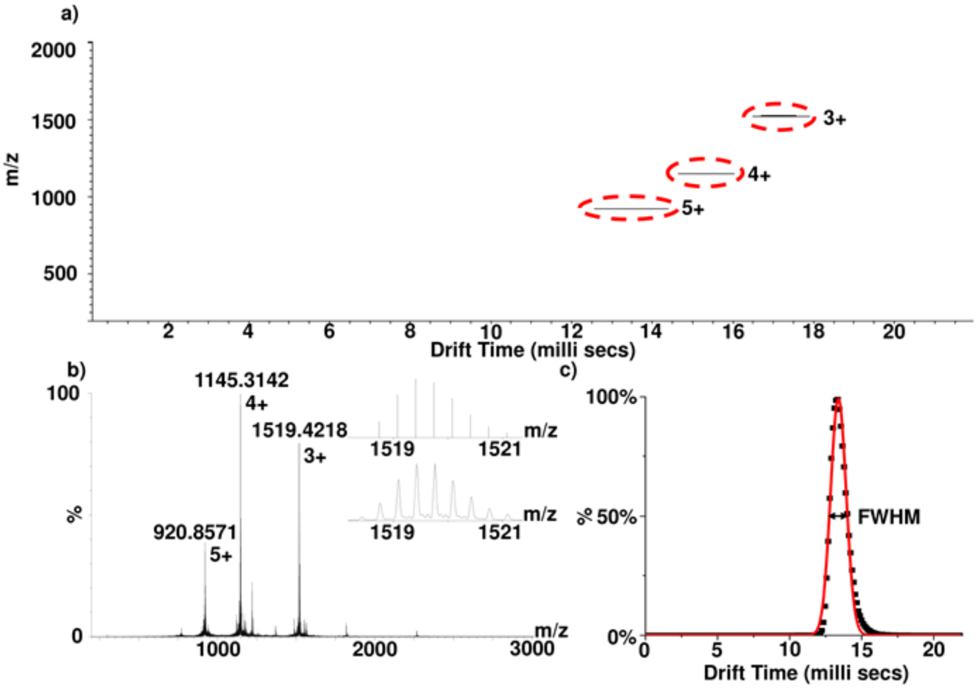

Figure S17. (a) IMS-MS spectrum; (b) isotope pattern and (c) drift-time distribution of [**4** + 3Na]$^{3+}$. The drift-time distribution was fitted by Gaussian normal distribution (red line) from the experimental data (black dots).

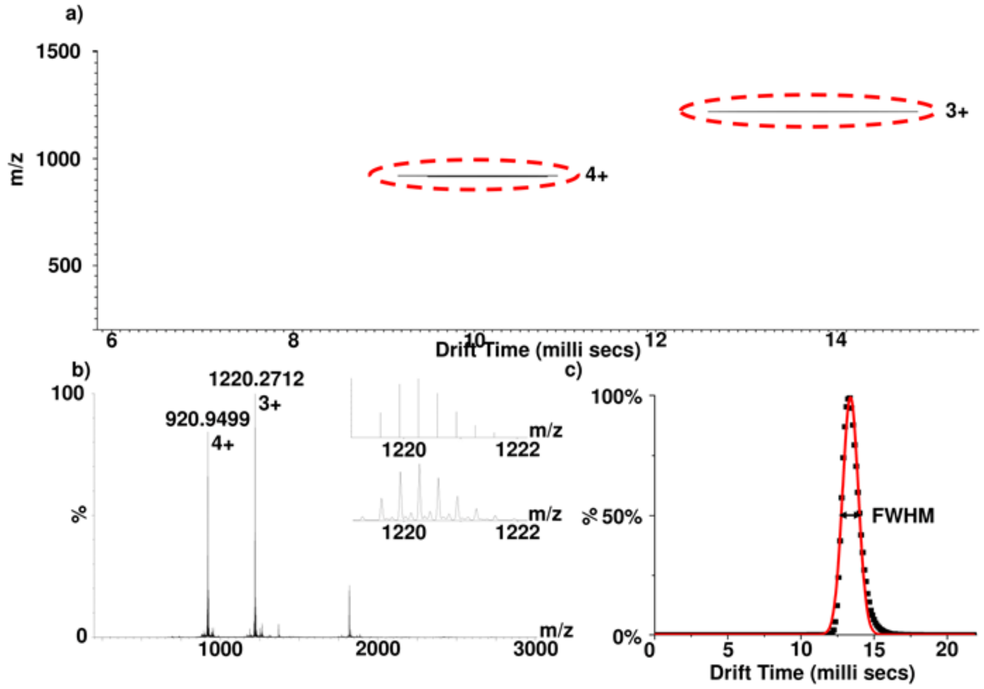

Figure S18. (a) IMS-MS spectrum; (b) isotope pattern and (c) drift-time distribution of [**5** + 3Na]$^{3+}$. The drift-time distribution was fitted by Gaussian normal distribution (red line) from the experimental data (black dots).

SPR sensorgrams

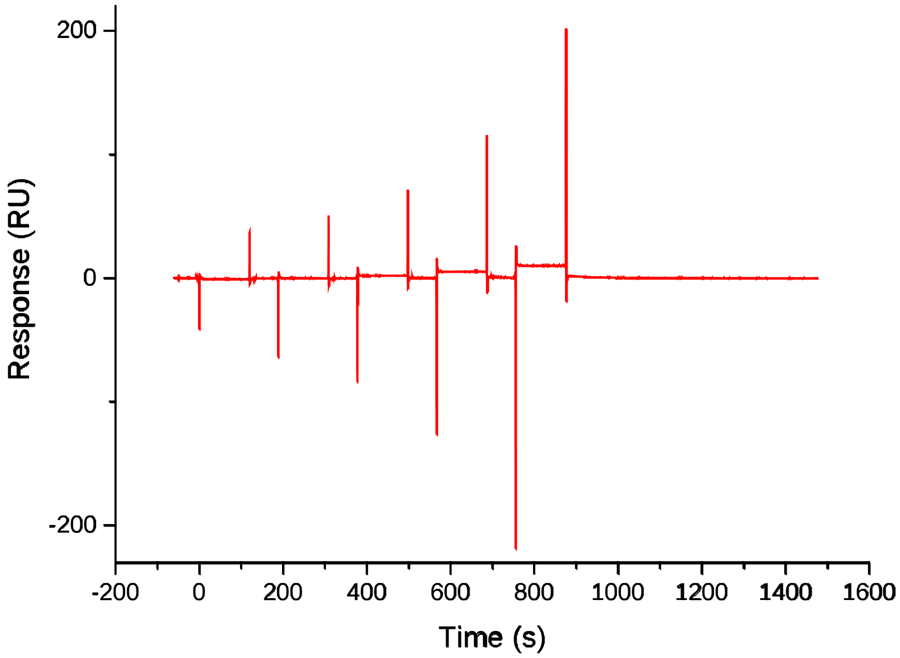

Figure S19. SPR sensorgram of Gb3-N_3. Measured at 62.5, 125, 250, 500, and 1000 μM with single-cycle kinetic experiment (cannot fit with Biacore software to provide kinetic parameters).
